# Quemliclustat and chemotherapy with or without zimberelimab in metastatic pancreatic adenocarcinoma: a randomized phase 1 trial

**DOI:** 10.1038/s41591-026-04283-z

**Published:** 2026-03-30

**Authors:** Zev A. Wainberg, Gulam A. Manji, Nathan Bahary, Susanna V. Ulahannan, Shubham Pant, David R. Spigel, Nataliya V. Uboha, Paul E. Oberstein, Anwaar Saeed, Brandon Beagle, Ji Yun Kim, Ning Wang, Ben Weeder, Shravani Shitole, Karim Mrouj, Jennifer R. Scott, Lisa G. Ensign, Daniel M. DiRenzo, Matthew J. Walters, Wilson Wu, Angelo Kaplan, Soonweng Cho, Omar Kabbarah, Eileen M. O’Reilly

**Affiliations:** 1 Department of Medicine, Division of Hematology and Oncology, David Geffen School of Medicine, University of California, Los Angeles, Los Angeles, CA USA; 2 Department of Medicine, Vagelos College of Physicians and Surgeons, Columbia University, New York, NY USA; 3 Department of Gastrointestinal Medical Oncology, Herbert Irving Comprehensive Cancer Center, Vagelos College of Physicians and Surgeons, Columbia University, New York, NY USA; 4 Department of Medical Oncology, Allegheny Health Network Cancer Institute, Pittsburgh, PA USA; 5 Hematology-Oncology Section, Department of Internal Medicine, Stephenson Cancer Center, College of Medicine, University of Oklahoma Health Sciences Center, Oklahoma City, OK USA; 6 Department of Gastrointestinal Medical Oncology, Division of Cancer Medicine, The University of Texas MD Anderson Cancer Center, Houston, TX USA; 7 Oncology Department, Sarah Cannon Research Institute, Nashville, TN USA; 8 Division of Hematology, Medical Oncology, and Palliative Care, Department of Medicine, University of Wisconsin, Madison, WI USA; 9 Carbone Cancer Center, Madison, WI USA; 10 Department of Medicine, Division of Hematology & Medical Oncology, NYU Langone Health, New York, NY USA; 11 Department of Medicine, Division of Hematology and Oncology, University of Pittsburgh Medical Center, Pittsburgh, PA USA; 12 Arcus Biosciences, Inc., Hayward, CA USA; 13 Medidata Solutions, Inc., a Dassault Systèmes company, New York, NY USA; 14 Gastrointestinal Oncology Service, Department of Medicine, Memorial Sloan Kettering Cancer Center, New York, NY USA; 15 Department of Medicine, Weill Cornell Medical College, New York, NY USA

**Keywords:** Cancer, Gastrointestinal diseases, Drug development, Gastrointestinal cancer, Immunotherapy

## Abstract

Quemliclustat potently inhibits CD73, a key enzyme producing immunosuppressive adenosine. In a phase 1b trial (ARC-8), we evaluated safety and efficacy of quemliclustat combined with gemcitabine/nab-paclitaxel (G/nP) with or without zimberelimab (anti-programmed cell death protein 1 (PD-1)) in first-line metastatic pancreatic ductal adenocarcinoma (PDAC). During the dose-escalation phase, 22 patients were enrolled across five dose levels of quemliclustat (25 mg, 50 mg, 75 mg, 100 mg or 125 mg) with G/nP + zimberelimab. During the dose-expansion phase, 116 patients were enrolled, beginning with a single-arm, non-randomized cohort receiving quemliclustat 100 mg + G/nP + zimberelimab, followed by a randomized cohort in which patients were assigned in a 2:1 ratio to receive quemliclustat 100 mg + G/nP with or without zimberelimab. The primary endpoint was safety and tolerability; secondary endpoints included assessments of clinical activity and survival. In all treatment arms, the safety profile was consistent with that of G/nP. Clinical response rates and survival outcomes were encouraging. *NR4A* family gene expression was upregulated by adenosine in vitro and by chemotherapy in human PDACs. High tumor *NR4A* expression was associated with improved overall survival (OS) in ARC-8 but not in two external cohorts from the PRINCE (G/nP + nivolumab (nivo)) or Morpheus-PDAC (G/nP) trials. Spatial tissue analyses revealed a scarcity of activated T cells near regions with high *NR4A1* expression, consistent with an immunosuppressed tumor microenvironment. In paired pretreatment/posttreatment biopsies, maximal downregulation of *NR4A* expression was associated with T cell activation and improved OS, pointing to a biological link between tumor adenosine and clinical benefit. ClinicalTrials.gov identifier: NCT04104672.

## Abstract (web summary)

In a phase 1b trial, patients with treatment-naive metastatic pancreatic adenocarcinoma received the CD73 inhibitor quemliclustat plus gemcitabine and nabpaclitaxel with or without the anti-PD1 antibody zimberelimab, showing encouraging clinical response rates and survival in quemliclustat-treated patients.

## Main

Patients with PDAC have an estimated 5-year survival rate of 13% with current therapies^1^. Metastatic PDAC (mPDAC) is largely resistant to standard-of-care cytotoxic chemotherapy combinations, with median OS of less than 1 year^2–7^. Multiple phase 3 trials of new therapeutic modalities have failed to improve on the standard care for mPDAC^3,8–13^. There is an urgent need for new therapies with novel mechanisms of action that exploit the uniquely aggressive pathobiology of mPDAC.

The ectonucleotidase CD73 is a key enzyme involved in the production of extracellular adenosine from adenosine triphosphate (ATP)^14^. Adenosine signaling protects healthy tissue from immune system activity, promotes angiogenesis and enhances wound healing^15,16^. Treatment of tumors with cytotoxic therapy, such as G/nP, leads to the release of ATP into the tumor microenvironment (TME), resulting in its CD73-mediated conversion into immunosuppressive adenosine^17,18^. Elevated adenosine levels in the TME suppress inflammation and immune function, markedly limiting the ability of the immune system to destroy tumor cells^18,19^. Quemliclustat is a potent and selective small‑molecule inhibitor of soluble and cell‑bound CD73 that is being studied in multiple tumor types^20,21^.

Molecular signatures reflective of cellular adenosine levels have been described^22,23^. However, these transcriptional signatures have limited predictive value, in part because they may not adequately reflect the cellular heterogeneity in the TME. Identification of gene expression profiles reflective of an adenosine-rich immunosuppressed TME^21,24–27^ may offer the potential to identify patients with cancer who could benefit from CD73 inhibition.

In the ARC-8 phase 1b study, we evaluated the safety and tolerability of quemliclustat combined with standard-of-care G/nP with or without the anti-PD-1 antibody zimberelimab, in patients with treatment-naive mPDAC. Our post hoc biomarker analysis focused on linking clinical benefit to adenosine biology and investigating the effects of quemliclustat treatment on the TME.

## Results

ARC-8 (NCT04104672) is an ongoing, phase 1b, open-label, dose-escalation and dose-expansion study conducted at 18 clinical sites in the United States. Patients were aged ≥18 years with a histologically or cytologically confirmed diagnosis of mPDAC, had no previous treatment for metastatic disease and had an Eastern Cooperative Oncology Group performance status (ECOG PS) of 0 or 1.

### Dose-escalation phase

**Fig. 1:**
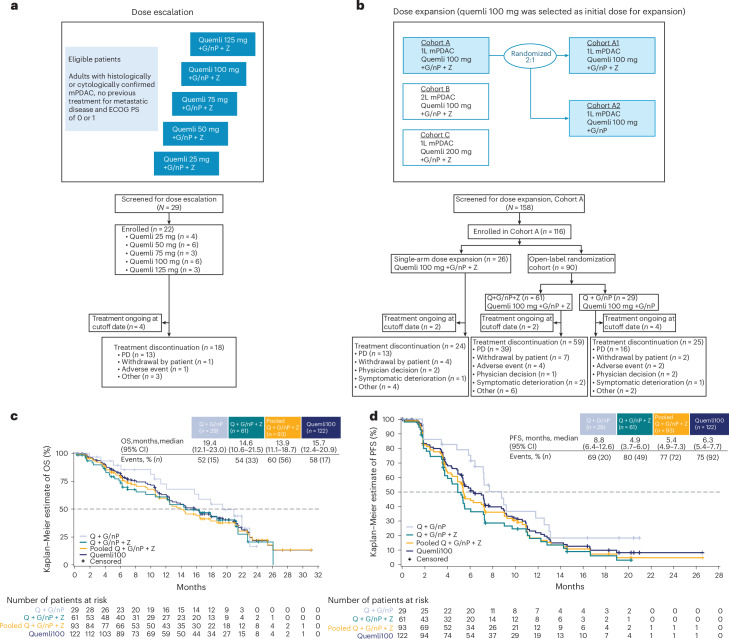
ARC-8 study design, patient flow and OS likelihoods. **a**, Dose-escalation paradigm including ECOG PS. Data cutoff date for the dose-escalation phase was 28 February 2022. **b**, Dose-expansion arms including Cohorts A, B and C. Data cutoff date for the dose-expansion phase was 19 June 2023. **c**, Kaplan−Meier plots of OS. **d**, Kaplan−Meier plots of PFS; 1L, first-line; 2L, second-line; PD, progressive disease; Q, quemliclustat 100 mg; Quemli100, all patients treated with Q + G/nP ± Z.

In the dose-escalation phase, 29 patients were screened and 22 patients were enrolled, starting on 3 February 2020 (first patient, first visit); four patients remained on treatment as of the data cutoff date of 28 February 2022 (Fig. 1a). Overall, 22 patients received quemliclustat combined with G/nP and zimberelimab, with quemliclustat doses of 25 mg (*n* = 4), 50 mg (*n* = 6), 75 mg (*n* = 3), 100 mg (*n* = 6) and 125 mg (*n* = 3). The median age (range) was 65 years (48–77) (Table 1).

**Table 1:** Patient demographics and characteristics

Dose-escalation phase	Quemli 25 mg + G/nP + Z (*n* = 4)	Quemli 50 mg + G/nP + Z (*n* = 6)	Quemli 75 mg + G/nP + Z (*n* = 3)	Quemli 100 mg + G/nP + Z (*n* = 6)	Quemli 125 mg + G/nP + Z (*n* = 3)	Overall (*N* = 22)
Age, years, median (range)	66 (64–69)	59 (48–77)	56 (53–56)	69 (52–76)	63 (52–77)	65 (48–77)
Sex, *n* (%)						
Male	2 (50)	4 (67)	2 (67)	3 (50)	1 (33)	12 (55)
Female	2 (50)	2 (33)	1 (33)	3 (50)	2 (67)	10 (45)
Race, *n* (%)						
White	4 (100)	4 (67)	2 (67)	4 (67)	2 (67)	16 (73)
Asian	0	1 (17)	1 (33)	1 (17)	0	3 (14)
Not reported	0	1 (17)	0	1 (17)	1 (33)	3 (14)
ECOG PS, *n* (%)						
0	4 (100)	4 (67)	3 (100)	3 (50)	1 (33)	15 (68)
1	0	2 (33)	0	3 (50)	2 (67)	7 (32)

Dose-expansion phase	Q + G/nP (*n* = 29)	Q + G/nP + Z (*n* = 61)	Pooled Q + G/nP + Z (*n* = 93)
Age, years, median (range)	65 (46–81)	66 (41–79)	66 (41–80)
Sex, *n* (%)			
Male	15 (52)	31 (51)	49 (53)
Female	14 (48)	30 (49)	44 (47)
Race, *n* (%)			
White	24 (83)	45 (74)	69 (74)
Asian	2 (7)	5 (8)	8 (9)
Black	1 (3)	4 (7)	5 (5)
Other or not reported	2 (7)	7 (11)	11 (12)
ECOG PS, *n* (%)			
0	9 (31)	19 (31)	33 (36)
1	20 (69)	42 (69)	60 (65)
Liver metastasis present at baseline^a^, *n* (%)	17 (59)	42 (69)	62 (67)
Prior pancreatic cancer surgery^b^, *n* (%)	8 (28)	7 (11)	13 (14)
Any prior systemic anticancer therapy, *n* (%)	4 (14)	6 (10)	11 (12)
Any prior radiotherapy, *n* (%)	1 (3)	4 (7)	9 (10)
Months since initial diagnosis^c^, median (range)	1.4 (0.2–49.2)	0.9 (0.1–54.6)	0.9 (0.1–54.6)
Ongoing treatment, *n* (%)	4 (14)	2 (3)	4 (4)
Discontinued all treatment, *n* (%)	25 (86)	59 (97)	89 (96)
Ongoing study follow-up, *n* (%)	11 (38)	18 (30)	26 (28)
Discontinued study, *n* (%)	18 (62)	43 (71)	67 (72)

^a^Derived from baseline tumor assessment data.

^b^Derived from prior procedure data.

^c^Stage not specified.

Pooled Q + G/nP + Z, all patients treated with Q and G/nP with Z; Q, quemliclustat 100 mg; quemli, quemliclustat.

As of the dose-escalation data cutoff date, all patients in the dose-escalation phase experienced at least one treatment-emergent adverse event (TEAE) (Table 2). One dose-limiting toxicity (DLT) of grade 2 autoimmune hepatitis occurred in the quemliclustat 50 mg cohort; this event resolved completely with steroid treatment, and the patient discontinued G/nP but continued to receive quemliclustat 50 mg and zimberelimab with no recurrence reported. No patients experienced a TEAE that resulted in death. Overall, more patients experienced grade 3 or higher TEAEs related to G/nP than to quemliclustat or zimberelimab. The selected recommended phase 2 dose (RP2D) of quemliclustat for expansion was 100 mg based on safety, tolerability, pharmacokinetics and pharmacodynamics, with no maximum tolerated dose identified.

**Table 2:** Adverse events

Dose escalation phase, n (%)	Quemli 25 mg +G/nP+Z (*n* = 4)	Quemli 50 mg +G/nP+Z (*n* = 6)	Quemli 75 mg +G/nP+Z (*n* = 3)	Quemli 100 mg +G/nP+Z (*n* = 6)	Quemli 125 mg +G/nP+Z (*n* = 3)	Overall (*N* = 22)
TEAE						
Any	4 (100)	6 (100)	3 (100)	6 (100)	3 (100)	22 (100)
Grade ≥3	3 (75)	6 (100)	2 (67)	5 (83)	3 (100)	19 (86)
SAE						
Any	1 (25)	4 (67)	2 (67)	4 (67)	0	11 (50)
Grade ≥3	1 (25)	4 (67)	1 (33)	4 (67)	0	10 (46)
Study drug-related TEAEs						
Any	4 (100)	6 (100)	3 (100)	6 (100)	3 (100)	22 (100)
Grade ≥3	3 (75)	6 (100)	1 (33)	5 (83)	2 (67)	17 (77)
Study drug-related SAEs						
Any	0	2 (33)	1 (33)	2 (33)	0	5 (23)
Grade ≥3	0	2 (33)	0	2 (33)	0	4 (18)
TEAEs grade ≥3 in ≥3 patients overall						
Anemia	0	4 (67)	1 (33)	3 (50)	1 (33)	9 (41)
Decreased neutrophil count	0	2 (33)	1 (33)	2 (33)	0	5 (23)
Decreased white blood cell count	0	1 (17)	1 (33)	3 (50)	0	5 (23)
Neutropenia	1 (25)	1 (17)	0	1 (17)	1 (33)	4 (18)
Decreased lymphocyte count	0	1 (17)	1 (33)	1 (17)	0	3 (14)
Decreased platelet count	1 (25)	2 (33)	0	0	0	3 (14)

Dose-Expansion Phase, n (%)	Q+G/nP (*n* = 29)	Q+G/nP+Z (*n* = 61)	Pooled Q+G/nP+Z (*n* = 93)
TEAEs			
Any	29 (100)	61 (100)	93 (100)
Study drug related	29 (100)	61 (100)	92 (99)
Grade ≥3	26 (90)	52 (85)	78 (84)
Immune mediated	2 (7)	6 (10)	10 (11)
Leading to study drug discontinuation	7 (24)	14 (23)	21 (23)
Leading to death	0	4 (7)	5 (5)
SAEs			
Any	15 (52)	29 (48)	50 (54)
Study drug related	10 (35)	14 (23)	24 (26)
Grade ≥3	13 (45)	24 (39)	42 (45)
Grade ≥3 study drug related	8 (28)	10 (16)	20 (22)
TEAEs grade ≥3 study drug related			
Any	22 (76)	45 (74)	67 (72)
Quemliclustat related	6 (21)	14 (23)	20 (22)
Zimberelimab related	0^a^	17 (28)	26 (28)
Gemcitabine related	19 (66)	43 (71)	64 (69)
Nab-paclitaxel related	21 (72)	43 (71)	64 (69)
TEAEs grade ≥3 study drug related occurring in ≥5% of patients			
Decreased neutrophil count	10 (35)	17 (28)	27 (29)
Anemia	8 (28)	14 (23)	20 (22)
Decreased white blood cell count	7 (24)	6 (10)	13 (14)
Decreased lymphocyte count	2 (7)	5 (8)	8 (9)
Fatigue	1 (3)	5 (8)	8 (9)
Sepsis	2 (7)	4 (7)	5 (5)

^a^Due to a data entry error, one patient in the Q + G/nP arm was originally reported as having a Z-related TEAE. The error was subsequently corrected.

ECOG PS, Eastern Cooperative Oncology Group performance status; G/nP, gemcitabine/nab-paclitaxel; Pooled Q + G/nP+Z, all patients treated with Q and G/nP with Z; Q, quemliclustat 100 mg; SAE, serious adverse event; TEAE, treatment-emergent adverse event; Z, zimberelimab.

### Dose-expansion phase

In the dose-expansion phase, 158 patients were screened and 116 were enrolled, starting on 5 January 2021 (first patient, first visit); eight patients remained on treatment at the dose-expansion data cutoff date of 19 June 2023 (Fig. 1b). In the randomized portion of the dose-expansion phase, patients were enrolled and randomized 2:1 to receive quemliclustat at RP2D combined with G/nP without zimberelimab (Q + G/nP arm; *n* = 29) or with zimberelimab (Q + G/nP + Z arm; *n* = 61). Baseline characteristics were similar between the two arms (Table 1). The Q + G/nP arm had approximately 10% fewer patients with liver metastases at baseline versus the Q + G/nP + Z arm (59% versus 69%). As of the dose-expansion data cutoff date, median survival follow-up was 21.1 months (95% confidence interval (CI): 19.8–22.3) for the Q + G/nP arm and 17.6 months (95% CI: 16.6–20.3) for the Q + G/nP + Z arm. In the Q + G/nP and Q + G/nP + Z arms, 14% and 3% of patients were ongoing treatment and 38% and 30% were ongoing study follow‑up, respectively.

#### Safety

Safety analyses were based on the safety-evaluable population, defined as all patients who received at least one dose of any study treatment. All patients in the randomized arms of the dose expansion reported at least one TEAE, most commonly fatigue, nausea and anemia (Table 2 and Supplementary Table 1). Approximately 10% of all patients reported immune-mediated TEAEs; 25% reported TEAEs leading to study discontinuation; and 50% reported at least one serious adverse event. No TEAEs leading to death were reported in the Q + G/nP arm, and four TEAEs leading to death were reported in the Q + G/nP + Z arm, with none related to quemliclustat or zimberelimab. The causes of the four deaths were respiratory failure (*n* = 2), sepsis (*n* = 1) and stroke (*n* = 1). Approximately 85% of patients in both arms reported at least one grade 3 or higher TEAE. Most reported grade 3 or higher TEAEs were related to G/nP rather than to quemliclustat or zimberelimab.

#### Efficacy

In the randomized arms, the confirmed objective response rate (ORR) was 38% (95% CI: 21–58) in the Q + G/nP arm and 25% (95% CI: 15–37) in the Q + G/nP + Z arm (Table 3). Unconfirmed ORR was 41% (95% CI: 24–61) in the Q + G/nP arm and 34% (95% CI: 23–48) in the Q + G/nP + Z arm. Confirmed disease control rate (DCR) was 86% (95% CI: 68–96) in the Q + G/nP arm and 72% (95% CI: 59–83) in the Q + G/nP + Z arm. Unconfirmed DCR was the same as confirmed DCR in both arms. Duration of response (DOR) was 5.5 months (95% CI: 4.1–11.2) in the Q + G/nP arm and 3.7 months (95% CI: 2.6–10.5) in the Q + G/nP + Z arm. Median OS was 19.4 months (95% CI: 12.1–23.0) in the Q + G/nP arm and 14.6 months (95% CI: 10.6–21.5) in the Q + G/nP + Z arm (Fig. 1c). Median progression-free survival (PFS) was 8.8 months (95% CI: 6.4–12.6) in the Q + G/nP arm and 4.9 months (95% CI: 3.7–6.0) in the Q + G/nP + Z arm (Fig. 1d).

**Table 3:** Clinical response

Dose-expansion phase, *n* (%)	Q + G/nP (*n* = 29)	Q + G/nP + Z (*n* = 61)	Pooled Q + G/nP + Z (*n* = 93)
ORR (95% CI)			
Confirmed	38% (21–58)	25% (15–37)	26% (17–36)
Unconfirmed	41% (24–61)	34% (23–48)	38% (28–48)
DCR (95% CI)			
Confirmed	86% (68–96)	72% (59–83)	75% (65–84)
Unconfirmed	86% (68–96)	72% (59–83)	75% (65–84)
DOR, months, median (95% CI)^a^	5.5 (4.1–11.2)	3.7 (2.6–10.5)	4.7 (3.3–9.3)

^a^Median was estimated using the Kaplan−Meier method, and 95% CI was calculated using the Brookmeyer−Crowley method.

Pooled Q + G/nP + Z, all patients treated with Q and G/nP with Z; Q, quemliclustat 100 mg.

### Post hoc analysis: efficacy of quemliclustat with or without zimberelimab

The Quemli100 cohort (*n* = 122) included all patients treated with quemliclustat 100 mg and G/nP with or without zimberelimab in the dose-escalation and dose-expansion phases; median survival follow-up was 21.0 months (95% CI: 19.0–22.8) as of the data cutoff date (19 June 2023). In the Quemli100 cohort, confirmed ORR was 29% (95% CI: 21–38), and unconfirmed ORR was 39% (95% CI: 30–48). Both confirmed and unconfirmed DCR was 78% (95% CI: 70–85). DOR was 5.4 months (95% CI: 3.7–9.3). Median OS was 15.7 months (95% CI: 12.4–20.9) (Fig. 1c). Median PFS was 6.3 months (95% CI: 5.4–7.7) (Fig. 1d). In the 43 (35%) patients without liver metastasis, median OS was 21.5 months (95% CI: 17.9–25.4), and, in the 79 (65%) patients with liver metastasis, median OS was 12.1 months (95% CI: 10.0–15.7) (Supplementary Table 2).

### Post hoc exploratory analysis: synthetic control arm comparison

**Extended Data Fig. 1:**
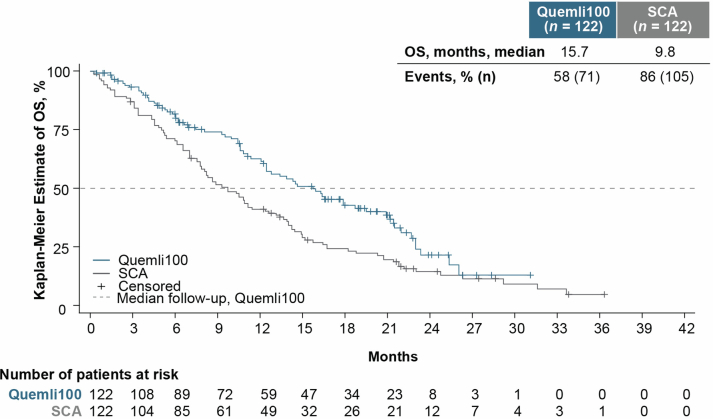
OS for the Quemli100 cohort versus SCA. G/nP, gemcitabine/nab-paclitaxel; OS, overall survival; Q, quemliclustat 100 mg; Quemli100, all patients treated with Q and G/nP with or without Z; SCA, synthetic control arm; Z, zimberelimab.

A total of 515 patients were identified from historical external global phase 2 and phase 3 mPDAC randomized clinical trials (approximately 50% from each phase). These patients received G/nP treatment alone and met the key eligibility criteria for the ARC-8 study (Supplementary Table 3). Of these 515 synthetic control arm (SCA)-eligible patients, 122 were 1:1 propensity score matched to the 122 patients in the Quemli100 cohort. SCA-eligible patients achieved an exact match with the Quemli100 patients for presence of liver metastases at baseline, as required, and also provided an exact match for ethnicity. The absolute standardized differences for all baseline covariates in the Quemli100 arm versus the SCA were ≤0.068, well below the target threshold of 0.25 (Extended Data Fig. 1 and Supplementary Table 4).

**Extended Data Fig. 2:**
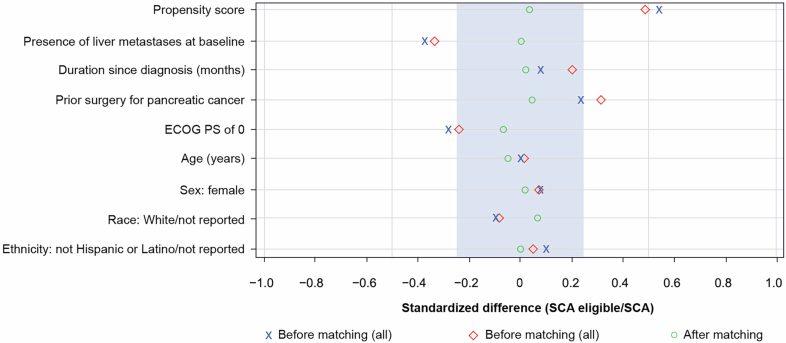
Standardized differences in baseline demographics and disease characteristics before and after matching in the SCA analysis. The shaded area indicates a standardized difference between −0.25 and 0.25. ECOG PS, Eastern Cooperative Oncology Group performance status; SCA, synthetic control arm.

Unconfirmed ORR was slightly lower numerically in the Quemli100 arm versus the matched SCA (39% (95% CI: 29.9–47.8) versus 41% (95% CI: 32.2–50.3); *P* = 0.794). Median PFS was not significantly different between the two arms (Quemli100, 6.3 months (95% CI: 5.4–7.7); SCA, 5.5 months (95% CI: 4.4–6.6); *P* = 0.110). Median OS was significantly longer in the Quemli100 arm (15.7 months (95% CI: 12.4–20.9)) versus the SCA (9.8 months (95% CI: 7.8–11.4)) (*P* = 0.003) (Extended Data Fig. 2).

### Post hoc tissue biomarker analyses: treatment-modulated adenosine in the pancreatic TME correlates with clinical benefit

#### *NR4A* family expression is upregulated by adenosine in the major cellular components comprising the TME

**Fig. 2:**
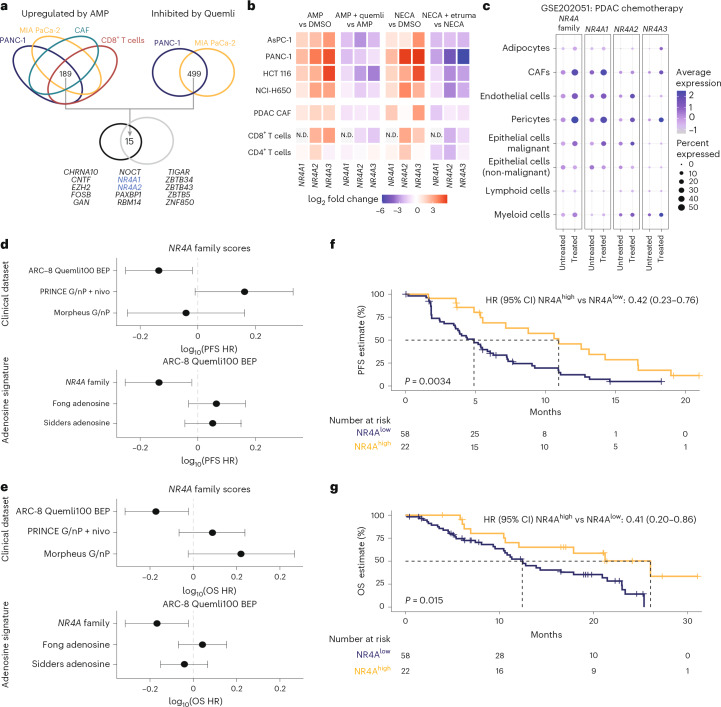
Expression of the *NR4A* gene family can be regulated by adenosine and is predictive of clinical benefit in ARC-8. **a**, Venn diagrams showing genes upregulated via RNA-seq experiments in four cell types comprising components of the TME (PANC-1, MIA PaCa-2, CAF and CD8^+^ T cells) and inhibited in two cell types (PANC-1 and MIA PaCa-2). A total of 15 genes were upregulated by AMP and inhibited by quemliclustat. Differentially expressed genes are defined as an increase or decrease of 50% log_2_ fold change with FDR ≤ 0.05. **b**, Heatmap of log_2_ fold change of RT−PCR values for *NR4A* family genes treated with adenosine-generating conditions or adenosine 2a receptor/adenosine 2b receptor agonist conditions and their corresponding inhibitors in expanded cell types in the TME. **c**, *NR4A* family genes can be upregulated to varying degrees in cell types comparing human PDAC in response to chemotherapy. **d**,**e**, Forest plots showing that high *NR4A* expression levels are predictive of improved PFS (**d**) and OS (**e**) in ARC-8 Quemli100 BEP but not in the PRINCE G/nP + nivo or MORPHEUS G/nP cohorts. Points represent the HR, and bars denote 95% CIs for each indicated comparison. Kaplan−Meier plots demonstrating improved PFS (**f**) and OS (**g**) likelihoods for patients with baseline tumors expressing high levels compared to low levels of *NR4A* genes by exploratory best cut analysis. *P* values were calculated using a log-rank test between groups. HRs and 95% CIs were calculated using Cox proportional hazards model. DMSO, dimethyl sulfoxide; etruma, etrumadenant (adenosine 2a receptor/adenosine 2b receptor antagonist); FDR, false discovery rate; HR, hazard ratio; N.D., not detected by RT−PCR; NR4A^high^, patients with high *NR4A* expression (*n* = 22); NR4A^low^, patients with low *NR4A* expression (*n* = 58); Q, quemliclustat 100 mg; Quemli100, all patients treated with Q + G/nP ± Z; quemli, quemliclustat.

**Extended Data Fig. 3:**
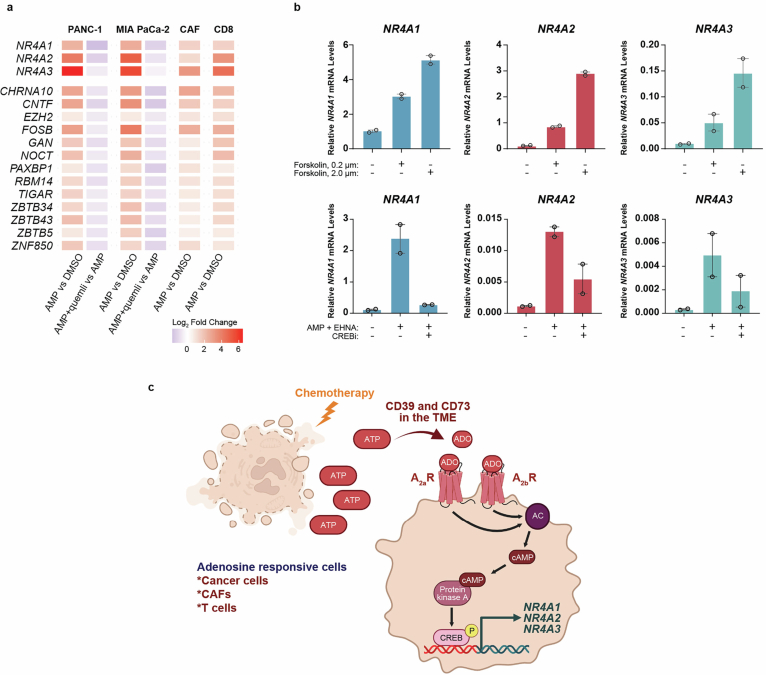
Genes regulated by adenosine in vitro. **a**, Upregulation of 13 genes and *NR4A* gene family by AMP in PANC-1 and MIA PaCa-2 cancer cell lines, as well as CAF and CD8^+^ T cell cultures, and reversal of gene expression in PANC-1 and MIA PaCa-2 cell lines by the addition of quemliclustat. **b**, Forskolin induces *NR4A1*, *NR4A2* and *NR4A3* expression through PKA in a dose-dependent manner, and AMP induced *NR4A* gene expression is prevented in the presence of a CREB inhibitor. Error bars denote SEM. **c**, A model for adenosine regulation of *NR4A* family expression. AMP, adenosine monophosphate; ATP, adenosine triphosphate; CAFs, cancer-associated fibroblasts; cAMP, cyclic adenosine monophosphate; CREB, cAMP response element binding protein; CREBi, CREB inhibitor; DMSO, dimethyl sulfoxide; EHNA, erythro-9-(2-hydroxy-3-nonyl)adenine; PKA, protein kinase A. Schematic in **c** created in BioRender; Direnzo, D. https://biorender.com/3dwgtsh (2025).

To investigate the quemliclustat mechanism of action, we began by identifying adenosine-responsive transcriptional changes across different cell types within the pancreatic TME, including cancer-associated fibroblasts (CAFs), CD8^+^ and CD4^+^ T cells and pancreatic cancer cell lines MIA PaCa-2 and PANC-1. For the cancer cell lines, experiments were conducted in the presence of quemliclustat, to prevent the conversion of adenosine monophosphate (AMP) to adenosine. Transcriptional profiling identified 189 genes that were upregulated by AMP across all cell types, and quemliclustat treatment resulted in transcriptional downregulation of 499 genes in MIA PaCa-2 and PANC-1 cells (Fig. 2a). Of the 15 intersecting genes with adenosine-dependent regulation (Fig. 2a and Extended Data Fig. 3a), two, *NR4A1* (*Nur77*) and *NR4A2* (*Nurr1*) (Fig. 2a), were members of the *NR4A* orphan nuclear receptor family implicated in T cell dysfunction^28–30^. *NR4A3* (*Nor1*) was also upregulated under all conditions (Extended Data Fig. 3a,b). Although quemliclustat inhibition of *NR4A3* upregulation did not reach significance, it was included in follow-up analyses. Upregulation of *NR4A* family expression after addition of AMP was confirmed and extended to additional cell types using reverse transcription polymerase chain reaction (RT−PCR), including colon cancer cell line HCT-116 and lung cancer cell line NCI-H650. Across all cell types, AMP and a stable adenosine analog, 5’-(N-ethylcarboxamido)adenosine (NECA), significantly upregulated all *NR4A* family members, which was inhibited by quemliclustat and the adenosine 2a receptor/adenosine 2b receptor antagonist etrumadenant, respectively (Fig. 2b).

Next, we assessed the effect of chemotherapy on *NR4A* family expression, leveraging public single-nucleus RNA sequencing (snRNA-seq) data from patients who underwent surgical resection with or without neoadjuvant chemotherapy treatment^31^. *NR4A* family members were upregulated to varying degrees in many cell types in the TME, including CAFs, as well as endothelial, pericyte, malignant epithelial and myeloid cells (Fig. 2c). Of note, *NR4A1* was expressed at high levels across many cell lineages and showed pronounced differential expression after treatment, reflecting, in part, generation of adenosine as a consequence of ATP release after cytotoxic killing of cancer cells^32^.

**Fig. 3:**
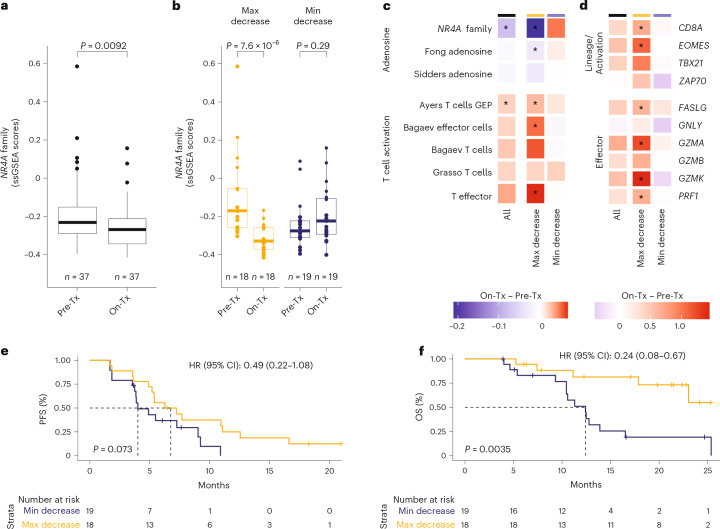
Expression of the *NR4A* family is downregulated by quemliclustat combination, and patients with the maximal downregulation have robust increase in T cell activity and receive the most survival benefit. **a**, Box plots of *NR4A* family ssGSEA scores in pretreatment and posttreatment samples of Quemli100 patients with 37 paired biopsies. **b**, Violin plots for *NR4A* family ssGSEA score change (posttreatment to pretreatment) split at the median into patient groups with maximal and minimal decrease. Horizontal lines represent the median; box spans the first and third quartiles and the fence (maximum whisker extension) at Q_1_ − IQR or Q_3_ + IQR; points (if any) outside of whiskers denote data spanning minima and maxima. **c**,**d**, Heatmaps showing ssGSEA score changes (posttreatment to pretreatment) across all patients (black) and groups defined in **b**, including patients with maximal (yellow) or minimal (blue) decrease in expression of *NR4A* family genes, for adenosine- and T cell-related gene sets (**c**) and genes from the Bagaev effector cells gene set (**d**). *P* values were calculated using paired Wilcoxon signed-rank test. *P ≤ 0.05. **e**,**f**, Kaplan−Meier curves and risk tables of PFS (**e**) and OS (**f**) of patients stratified by minimal and maximal *NR4A* family decrease, defined in **b**. Dotted lines denote median survival. *P* values were calculated using the log-rank test. HRs and 95% CIs were calculated using Cox proportional hazards regression. HR, hazard ratio; max, maximum; min, minimum; IQR, interquartile range; Q, quemliclustat 100 mg; Quemli100, all patients treated with Q + G/nP ± Z; Tx, treatment.

We then investigated a potential mechanism of *NR4A* family regulation through the cyclic AMP (cAMP)–protein kinase A (PKA)–cAMP-responsive element-binding protein (CREB) pathway^33,34^ downstream of adenosine receptor activation. Treatment of PANC-1 cells with forskolin, an adenylyl cyclase activator, resulted in dose-dependent upregulation of all three *NR4A* family members (Extended Data Fig. 3b). PANC-1 cells treated with AMP and erythro-9-(2-hydroxy-3-nonyl)adenine (EHNA) showed significant *NR4A* family upregulation, whereas co-treatment with a CREB inhibitor^35,36^ reversed adenosine-mediated induction to near-baseline levels (Extended Data Fig. 3b). These results suggest that adenosine can regulate *NR4A* expression through the cAMP−PKA−CREB signaling axis, establishing *NR4A* family members as downstream targets of adenosine biology (Fig. 3c).

#### Baseline tumor *NR4A* family expression is predictive of improved clinical benefit in ARC-8

**Extended Data Fig. 4:**
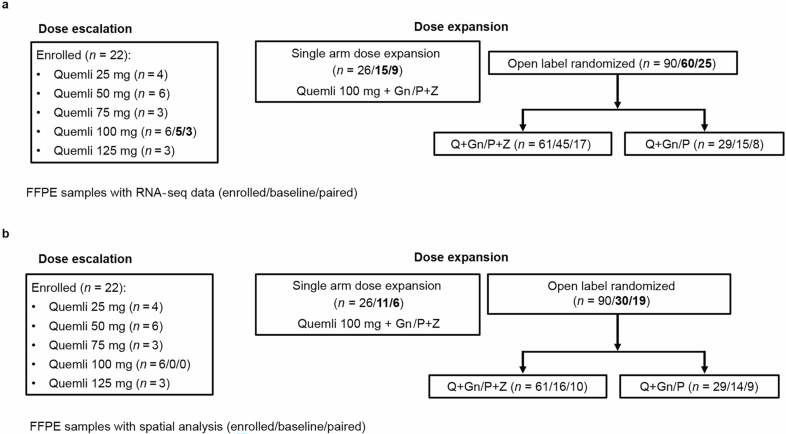
Breakdown of available tissue samples. **a**, RNA-seq and **b**, spatial analysis from the ARC-8 study. FFPE, formalin-fixed paraffin-embedded; G/nP, gemcitabine/nab-paclitaxel; Quemli, quemliclustat; RNA-seq, RNA sequencing; Z, zimberelimab.

We examined the association between *NR4A* family expression and clinical outcomes in patients with available transcriptomic data from first-line PDAC clinical cohorts, including from patients randomized to the G/nP + nivo arm of the PRINCE trial (PRINCE G/nP + nivo)^12^ and patients randomized to the G/nP arm of the MORPHEUS-PDAC trial (MORPHEUS G/nP)^37^ (Fig. 2c,d). We also performed transcriptome sequencing on baseline tumor tissues from 80 patients treated as part of the Quemli100 cohort from ARC-8, termed the biomarker evaluable population (BEP) (Extended Data Fig. 4a). The clinical characteristics of the BEP were similar to those of the overall ARC-8 patient cohort (Supplementary Table 5). The *NR4A* family expression significantly correlated with survival benefit in the BEP (PFS: hazard ratio = 0.732 (95% CI: 0.56–0.96); *P* = 0.0238; OS: hazard ratio = 0.678 (95% CI: 0.49–0.95); *P* = 0.0239) but not in the PRINCE G/nP + nivo or MORPHEUS G/nP clinical cohorts (Fig. 2d,e). By contrast, previously published adenosine signatures^22,23^ were not associated with OS or PFS (Fig. 2d,e). These findings suggest that *NR4A* family expression is predictive of patient benefit from a quemliclustat-containing regimen rather than prognostic of general mPDAC outcomes.

**Extended Data Fig. 5:**
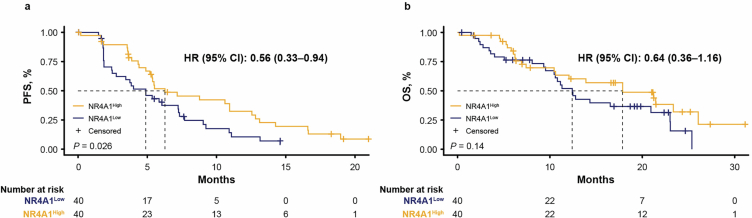
ARC-8 KM survival probability analysis by *NR4A* level by median cut. a. PFS and b. OS. *P* values were calculated using log-rank test. HR and CI were calculated using Cox proportional hazards model. CI, confidence interval; HR, hazard ratio; KM, Kaplan−Meier; OS, overall survival; PFS, progression-free survival.

**Extended Data Fig. 6:**
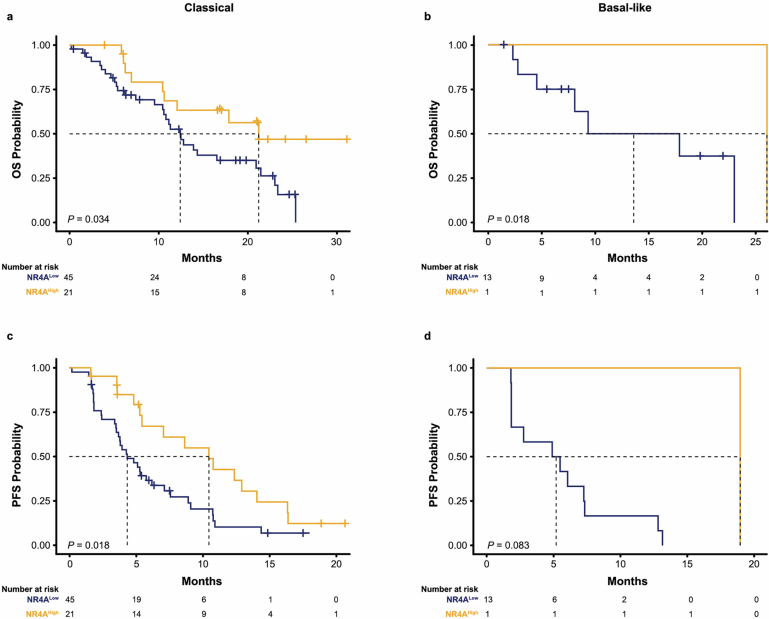
*NR4A* status predicts clinical benefit in classical and basal-like molecular subtypes. ARC-8 KM for **a**, **c**, classical molecular subtype (*n* = 66) and **b**, **d**, basal-like molecular subtype (*n* = 14) using *NR4A* bestcut analysis of the ARC-8 BEP. *P* values were calculated using log-rank test. BEP, biomarker evaluable population; KM, Kaplan−Meier; OS, overall survival; PFS, progression-free survival.

Patients with high *NR4A* expression (NR4A^high^ group; *n* = 22) showed significantly improved PFS compared to those with low expression (NR4A^low^ group; *n* = 58) by exploratory best cut analysis (hazard ratio = 0.42 (95% CI: 0.23–0.76); *P* = 0.0034) (Fig. 2f). The median PFS for the NR4A^high^ group was 11.0 months compared to 4.9 months for the NR4A^low^ group. Similarly, OS was significantly longer for the NR4A^high^ group (hazard ratio = 0.41 (95% CI: 0.20–0.86); *P* = 0.015) (Fig. 2g). The median OS was 26.1 months for the NR4A^high^ group compared to 12.4 months for the NR4A^low^ group. Similar trends were observed when a median cutoff was applied (Extended Data Fig. 5). We classified patients in the ARC-8 BEP into classical molecular subtype (*n* = 66 (83%)) or basal-like molecular subtype (*n* = 14 (18%))^38^ and found that *NR4A* status may portend improved clinical benefit in both settings (Extended Data Fig. 6).

**Extended Data Fig. 7:**
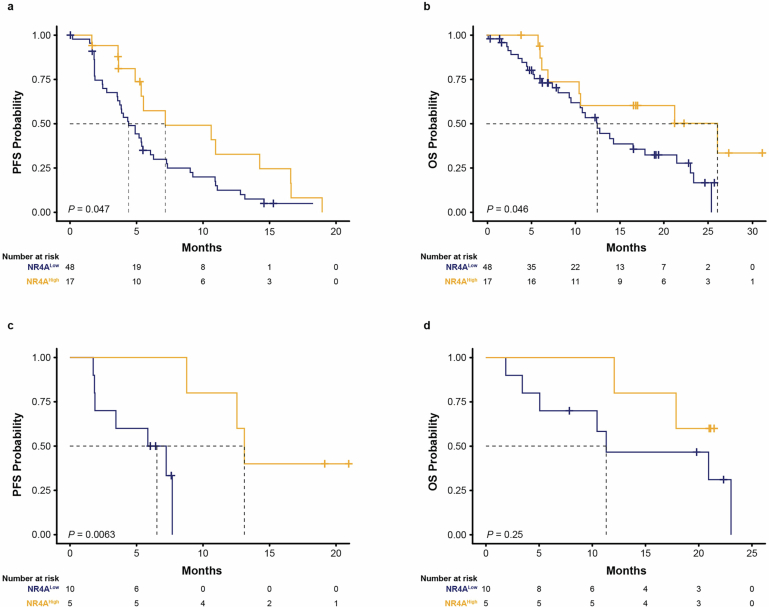
ARC-8 KM using *NR4A* bestcut analysis of the ARC-8 BEP. **a**, **b**, Q + G/nP+Z (*n* = 65) and **c**, **d**, Q + G/nP (*n* = 15). *P* values were calculated using log-rank test. BEP, biomarker evaluable population; G/nP, gemcitabine/nab-paclitaxel; KM, Kaplan−Meier; OS, overall survival; PFS, progression-free survival; Q, quemliclustat 100 mg; Z, zimberelimab.

To begin to assess the potential contribution of zimberelimab to these observations, we compared the clinical outcomes of NR4A^high^ and NR4A^low^ groups in the Q + G/nP + Z (*n* = 65) and Q + G/nP (*n* = 15) arms of the BEP. We observed similar PFS and OS trends in both arms (Extended Data Fig. 7), suggesting that the presence of zimberelimab did not contribute meaningfully to the predictive value of *NR4A* family expression and that this signature is likely predictive of clinical benefit of quemliclustat.

#### A scarcity of activated T cells in proximity to NR4A1^high^ regions in the TME

**Extended Data Fig. 8:**
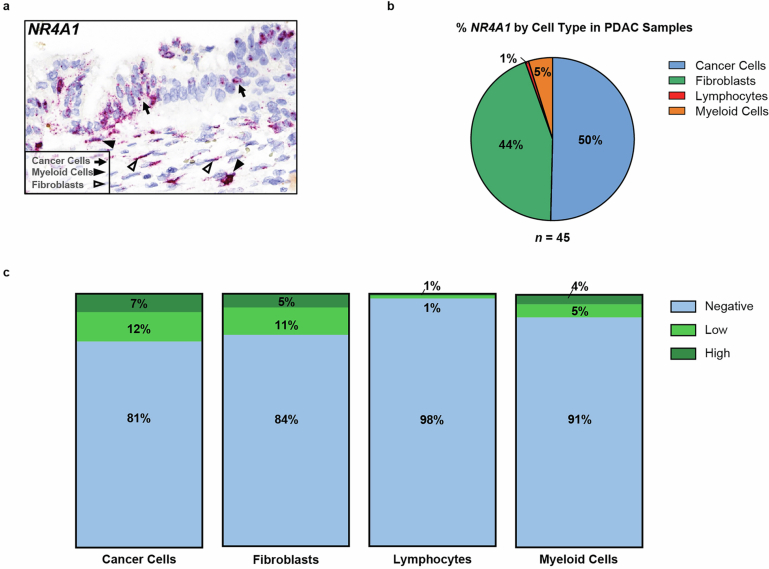
*NR4A1* is expressed in multiple cell types within the tumor microenvironment in tumor tissues from ARC-8 (n = 71 biopsies). **a**, *NR4A1* expression by ISH (red chromogen) is observed in cancer cells (arrows), myeloid cells (solid arrowheads) and fibroblasts (open arrowheads). **b**, Pie chart depicting *NR4A1* expression distribution in cell types that comprise the tumor microenvironment. **c**, Proportions of *NR4A1* negative, low and high expressing cell types comprising the PDAC tumor microenvironment. ISH, in situ hybridization; NR4A1^high^, cells with *NR4A*1 > 16 copies; NR4A1^low^, cells with < 4 *NR4A1* copies; NR4A1^negative^, cells with no *NR4A1* copies; PDAC, pancreatic ductal adenocarcinoma.

We evaluated the spatial expression patterns of *NR4A* family members in tissue samples from ARC-8 patients using dual *NR4A1/IFNγ* in situ hybridization (ISH). A total of 71 tumor samples were assessed (Extended Data Fig. 4b). ISH protocols for *NR4A1*, *NR4A2* and *NR4A3* were developed, and *NR4A1* was the most abundantly expressed family member in multiple cell types in the TME (Extended Data Fig. 8). Additionally, a multiplex immunofluorescence (mIF) assay was deployed on tumor tissues to quantify the numbers and spatial distribution of cytotoxic, regulatory and exhausted T cells in the TME.

**Extended Data Fig. 9:**
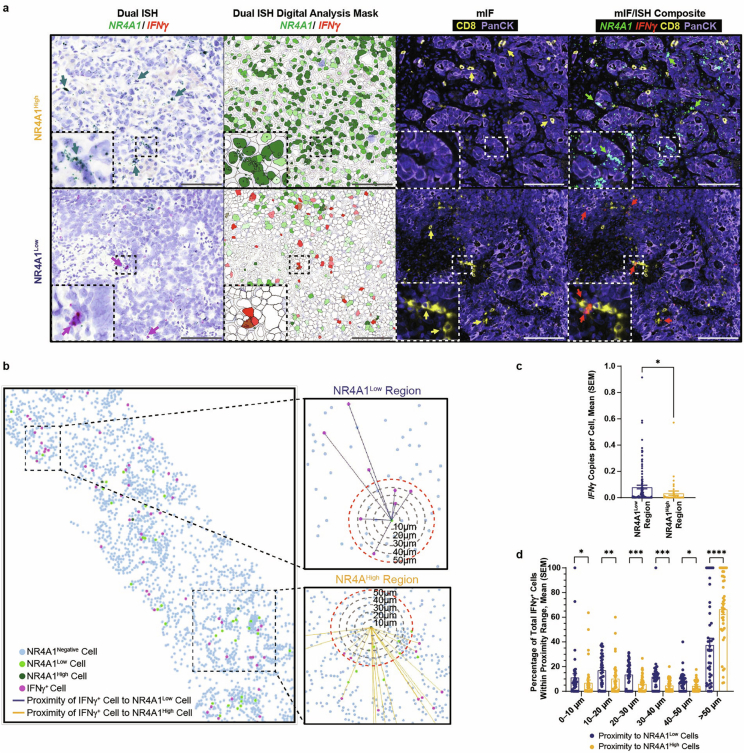
Regions of high *NR4A1* expression create an immunosuppressive gradient in the tumor microenvironment. **a**, Photomicrographs of dual ISH for *NR4A1* (green) and *IFNγ* (red) with digital analysis overlay showing low levels of *IFNγ* expression when *NR4A1* expression is high (top panel) versus high levels of *IFNγ* expression when *NR4A1* is low (bottom panel) (*n* = 41 biopsies). mIF images and fused mIF + ISH images on the right confirm the presence of CD8^+^ T cells (yellow) expressing *IFNγ* (red) when *NR4A1* is low compared to CD8^+^ T cells with no *IFNγ* expression when *NR4A1* is high (green). Scale bar, 100 µm. **b**, Spatial plot showing NR4A1^low^ copy number cells (light green), *NR4A1*^high^ copy number cells (dark green) and IFNγ^+^ cells (pink) with insets of *NR4A1*^low^ and *NR4A1*^high^ ROIs. The top ROI inset shows nearest neighbor proximity lines of an NR4A1^low^ cell in close proximity to IFNγ^+^ cells (blue proximity lines), whereas the bottom ROI shows an NR4A1^high^ cell with IFNγ^+^ cells located further away (gold proximity lines). Concentric circles demonstrate the proportion of IFNγ^+^ cells at distances within 50 μm. **c**, Digital quantification of *IFNγ* and *NR4A1* transcripts in T cell enriched ROIs (*n* = 150 ROIs from 41 biopsies) shows significantly lower *IFNγ* expression (as defined by average copies of *IFNγ* per cell) in NR4A1^high^ regions (*P* = 0.0367). **d**, Proximity analysis shows a significantly higher proportion of IFNγ^+^ cells are located within 10, 20, 30, 40 and 50 μm distances of NR4A1^low^ cells compared to NR4A1^high^ cells, while a higher percentage of IFNγ^+^ cells are located > 50 μm from NR4A1^high^ cells compared to NR4A1^low^ cells (*n* = 41 biopsies) (*P* = 0.0418, 0.0084, 0.0008, 0.0004, 0.0393 and < 0.0001, respectively). The bars in **c** and **d** represent the mean values ± SEM. *P* values were calculated using nonparametric two-sided Mann-Whitney *t*-tests. *P ≤ 0.05, **P ≤ 0.01, ***P ≤ 0.001, ****P ≤ 0.0001. IFNγ^+^, cells positive for IFNγ regardless of the number of copies; IFN, interferon; ISH, in situ hybridization; mIF, multiplex immunofluorescence; NR4A1^high^, cells with *NR4A1* > 16 copies; NR4A1^low^, cells with *NR4A1* < 4 copies; NR4A1^negativ^^e^, cells with no *NR4A1* copies; ROI, regions of interest.

**Extended Data Fig. 10:**
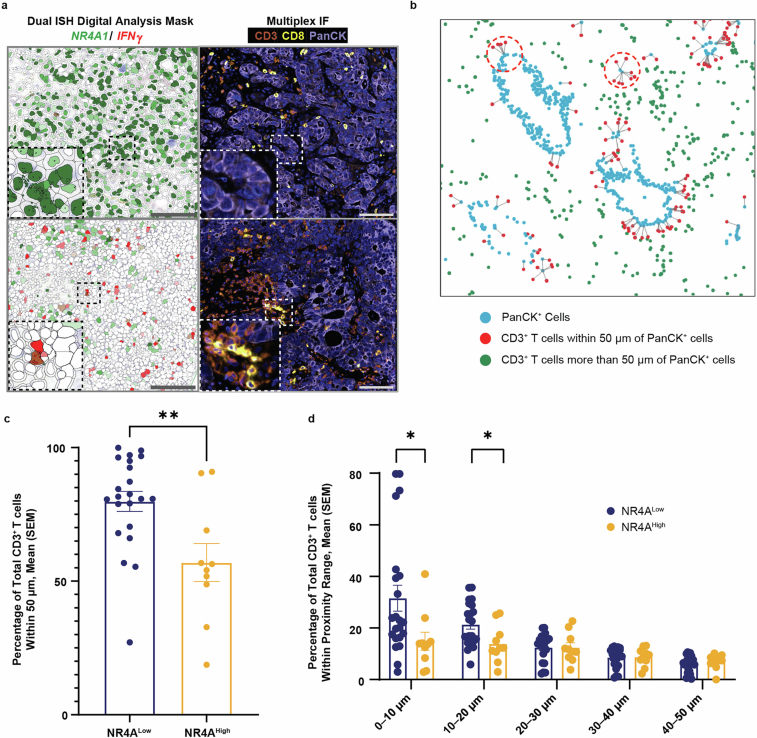
Adenosine induces regions of high NR4A expression that create an immunosuppressive gradient in the tumor microenvironment. **a**, Dual ISH for *NR4A1* (green) and *IFNγ* (red) with digital analysis overlay showing low levels of *IFNγ* expression when *NR4A1* expression is high (top panel) versus high levels of *IFNγ* expression when *NR4A1* is low (bottom panel). mIF images on the right confirm the presence of high numbers of CD3^+^ T cells (maroon), including CD8^+^ T cells (yellow), in the areas with *IFNγ* expression (red) when *NR4A1* is low (bottom panels) compared with lower numbers of CD3^+^ T cells with no *IFNγ* expression when *NR4A1* is high (top panels) (n = 41 biopsies). Scale bar, 100 µm. **b**, Proximity analysis plot showing CD3^+^ T cells within 50 μm (red) of PanCK+ cancer cells (blue) and CD3^+^ T cells more than 50 μm (green) away from PanCK^+^ cells. **c**, Quantification of %CD3^+^ T cells within 50 μm proximity to PanCK^+^ cancer cells shows significantly lower levels of CD3^+^ T cells within 50 μm to cancer cells in the setting of high *NR4A* expression by RNA-seq compared with samples with low *NR4A* levels (*P* = 0.0055) (n = 32 biopsies). **d**, Analysis of T cell frequency at 10 μm intervals from cancer cells (defined by PanCK) shows that a larger proportion of T cells were localized within 0–10 μm and 10–20 μm of cancer cells in tumors with low *NR4A* than in tumors with high *NR4A*, but further distances were not significantly different (*P* = 0.01406 and 0.01699, respectively) (n = 32 biopsies). The bars in c and d represent the mean values +/- SEM. *P* values were calculated using nonparametric two-sided Mann-Whitney *t*-tests. *P ≤ 0.05, **P ≤ 0.01. IF, immunofluorescence; IFN, interferon; ISH, in situ hybridization; mIF, multiplex immunofluorescence; RNA-seq, RNA sequencing.

We assessed the spatial distribution of *NR4A1*-expressing cells and activated T cells, as indicated by *IFNγ* transcript numbers, in 150 different T-cell-enriched regions of interest (ROIs) and determined a median T cell content of 329.8 CD3^+^ cells per mm^2^ and 78.4 CD8^+^ T cells per mm^2^. Using the 75th percentile of average *NR4A1* copies per cell as a threshold, we categorized *NR4A1* transcript numbers into high and low status. There were significantly fewer *IFNγ* transcripts per cell in NR4A1^high^ versus NR4A1^low^ ROIs (Extended Data Fig. 9a−c). To confirm that the *IFNγ* finding was indeed a measurement of IFNγ^+^CD8^+^ T cells, fused images were generated, combining the ISH-stained and mIF-stained serial sections. The analysis showed that IFNγ^+^CD8^+^ T cells and *NR4A1* transcripts were largely co-localized in the vicinity of cancer cells (Extended Data Figs. 9a and 10).

Next, we conducted proximity analysis to quantify the number of T cells in close proximity to cancer cells, which are the predominant source of *NR4A1* expression in the TME (Extended Data Fig. 10). Interestingly, NR4A^high^ tumors (*n* = 10) showed significantly fewer T cells within 50 µm of PanCK^+^ cancer cells compared to NR4A^low^ tumors (*n* = 22), with a mean of 57% of total T cells versus 80%, respectively (*P* = 0.0055) (Extended Data Fig. 10). This difference was driven by T cells in the range of 0–10 µm and 10–20 µm, which were scarce in NR4A^high^ tumors compared to NR4A^low^ tumors, with means of 15% versus 32% within 10 µm (*P* = 0.0141) and 14% versus 21% within 10–20 µm (*P* = 0.0170), respectively (Extended Data Fig. 10). This finding was also demonstrated by an average distance between CD3^+^ to PanCK^+^ cells of 66 µm in NR4A^high^ tumors versus 33 µm in NR4A^low^ tumors (*P* = 0.0066) (Extended Data Fig. 10).

Expanding on the spatial distribution of T cells in relation to an immunosuppressed TME, nearest neighbor and proximity analyses were performed using *NR4A1* transcript number and localization as an indicator of adenosine levels and *IFNγ* as a marker of T cell activation and inflammation (Extended Data Fig. 9b). Concentric circles drawn around NR4A1^high^ and NR4A1^low^ regions in the TME were used to quantify the percentage of IFNγ^+^ cells within 10-µm intervals of *NR4A1*-expressing cancer cells (Extended Data Fig. 9b). The percentage of total IFNγ^+^ cells located across 10-µm intervals within a 50-µm distance from NR4A1^high^ cells was significantly lower than that observed for NR4A1^low^ cells (Extended Data Fig. 9d). Spatial analyses showed that IFNγ^+^ T cells were distributed significantly further away from cells expressing high levels of *NR4A1* (67% of IFNγ^+^ cells located >50 µm away) compared to cells with low *NR4A1* expression (37% of IFNγ^+^ cells located >50 µm away) (*P* < 0.0001) (Extended Data Fig. 9d). This spatial relationship was also reflected in IFNγ^+^ T cell average distance measurements of 74.6 µm relative to cells with NR4A1^high^ copy numbers versus 40.9 µm relative to NR4A1^low^ regions (*P* = 0.0171). These findings support the presence of an adenosine immunosuppressive gradient effect that may contribute to a scarcity of IFNγ^+^ T cells in close proximity to cancer cells expressing high levels of *NR4A* in the TME.

#### Treatment with quemliclustat regimens in ARC-8 results in a decrease in tumor *NR4A* expression and an increase in T cell activation in the TME

To determine if a quemliclustat regimen was capable of downregulating *NR4A* expression in patients, we assessed *NR4A* transcript levels in 37 paired pretreatment/posttreatment tumor samples from the Quemli100 cohort using RNA-seq (Extended Data Fig. 4a and Supplementary Table 5). We found that, compared to pretreatment levels, tumor *NR4A* expression was significantly downregulated posttreatment with quemliclustat (*P* = 0.0092) (Fig. 3a). Paired samples were stratified into ‘maximal decrease’ and ‘minimal decrease’ subgroups based on the median cut of *NR4A* expression change after treatment (Fig. 3b). We evaluated several T cell activation signatures^39–41^ in the paired samples and observed significant upregulation of multiple signatures in the maximal decrease subgroup but not in the minimal decrease subgroup (Fig. 3c). Gene-level analysis revealed significant upregulation of T cell lineage markers CD8A and EOMES as well as the effector molecules FASLG, GZMA, GZMK and PRF1 exclusively in the *NR4A* maximal decrease subgroup (Fig. 3d). These findings suggest that high-magnitude *NR4A* downregulation after treatment may be associated with the degree of T cell activation, potentially shedding light on a mechanism of action of quemliclustat.

#### Greater magnitude of reduction in tumor *NR4A* expression after treatment is associated with improved OS in ARC-8

Next, we investigated the clinical importance of tumor *NR4A* family downregulation after treatment in the paired pretreatment/posttreatment biopsies. We performed Kaplan−Meier analyses comparing survival outcomes in the *NR4A* maximal and minimal decrease subgroups after quemliclustat treatment. Maximal decrease in *NR4A* family expression after treatment was associated with a positive trend toward improved PFS (hazard ratio = 0.49 (95% CI: 0.22–1.08); *P* = 0.073) and was significantly associated with improved OS (hazard ratio = 0.24 (95% CI: 0.08–0.67); *P* = 0.0035) (Fig. 3e,f). OS benefit was notable in the *NR4A* maximal decrease subgroup, with more than 50% of patients still on treatment after more than 25 months of follow-up (Fig. 3f). Median OS was close to 12 months even in the *NR4A* minimal decrease subgroup, longer than the 9.8 months shown for the SCA group. These results demonstrate that the magnitude of *NR4A* family expression downregulation after treatment may be predictive of clinical outcomes in ARC-8 and further supports a link between clinical benefit on quemliclustat and adenosine modulation in the TME.

## Discussion

Quemliclustat targets the adenosine-rich immunosuppressive TME by inhibiting CD73, reducing adenosine-regulated gene expression and increasing inflammation in a novel approach to treating patients with mPDAC^5,42^. In this phase 1b study, an RP2D of quemliclustat 100 mg was established and used to treat 116 patients with therapy-naive mPDAC in combination with standard-of-care G/nP chemotherapy with or without zimberelimab. The safety profile was similar to historical data for G/nP alone regarding overall TEAEs, grade 3 or higher TEAEs, serious adverse events and TEAEs leading to death^7,43^. In all groups, the highest proportion of patients experienced TEAEs related to chemotherapy rather than those associated with quemliclustat or zimberelimab.

Most pancreatic tumors possess a mutation in *KRAS*, and tumors harboring mutations in the RAS signaling pathway express increased levels of CD73 (refs. ^26,27^). High CD73 expression is linked to perineural invasion, lymph node metastasis and poor survival^25^. In preclinical models, CD73 inhibition drives enhanced cytotoxic T cell activity and tumor control^21^. Results from the ARC-8 study support CD73 as a therapeutic target in PDAC, which is being further investigated in the phase 3 PRISM-1 trial (NCT06608927) comparing quemliclustat and chemotherapy to placebo and chemotherapy^44^.

Although direct comparisons across clinical trials should be approached with caution, the median OS of 15.7 months in the Quemli100 cohort compares favorably with the median OS of historical benchmarks of G/nP alone, including the phase 3 NAPOLI 3 study (median OS, 9.2 months) and the registrational phase 3 MPACT study (median OS, 8.7 months)^7,45^. In a phase 1b/2 randomized study of the CD73 inhibitor oleclumab plus durvalumab added to G/nP, the median OS was 12.9 months, which was not significantly different from the median OS of 10.8 months that was observed with G/nP alone (hazard ratio = 0.75 (95% CI: 0.50–1.13))^46^. Improvements in OS can be influenced by subsequent treatments, such as KRAS inhibitors, but just two patients in ARC-8 received subsequent KRAS protein degrader, and none received KRAS inhibitors.

The post hoc SCA exploratory analysis was designed to overcome the limitations of traditional unadjusted historical controls by careful selection and prespecification of historical data and alignment of patient-level characteristics. While blinded to all patient-level outcomes, SCA-eligible patients were selected from one of four randomized controlled global clinical trials of mPDAC, which were completed as recently as 2023. Propensity score matching, including exact matching on the presence of liver metastases at baseline, was used to reduce the potential for confounding and to create an SCA that was well balanced to the baseline demographics and clinical characteristics of patients in the Quemli100 cohort. Compared to the SCA, patients treated with the quemliclustat combinations demonstrated an increase in median OS of 5.9 months (hazard ratio = 0.634 (95% CI: 0.471–0.854); *P* = 0.003) and a trend in improvement of PFS with a 22% reduction in the risk of progression or death.

Two transcriptionally defined signatures reflective of tumor adenosine levels were previously reported^22,23^. Assessment of these signatures in paired pretreatment/posttreatment tumor biopsies from ARC-8 did not demonstrate meaningful modulation or a predictive value. Consequently, we identified *NR4A* family expression signatures as being regulated by adenosine in vitro, increased in various cellular components of the TME in human PDACs after cytotoxic chemotherapy and downregulated after treatment in tumors from ARC-8. Thus, an *NR4A* expression signature may represent a reasonable surrogate for assessing adenosine levels in the TME.

Our findings suggest that clinical outcomes in ARC-8 may be linked to adenosine-regulated *NR4A* family expression levels in baseline tumors. Patients with NR4A^high^ tumors had significantly improved PFS and OS compared to those with NR4A^low^ mPDAC (PFS: hazard ratio = 0.732; OS: hazard ratio = 0.678). Notably, the *NR4A* signature did not demonstrate a survival advantage in either the PRINCE G/nP + nivo or the MORPHEUS G/nP cohorts, providing further support that the clinical benefit observed in ARC-8 was likely driven, at least in part, by quemliclustat.

From our spatial analysis, we identified a potential immunosuppressive gradient effect for NR4A^high^ regions on the numbers and localization of IFNγ^+^ T cells in the TME. We observed fewer T cells and a scarcity of IFNγ^+^ T cells within less than 50 µm and a higher abundance of IFNγ^+^ T cells at distances of more than 50 µm from NR4A^high^ tumor cells. Hence, high levels of adenosine-regulated *NR4A* expression may be reflective of an immunosuppressed TME with few activated cytotoxic T cells in the vicinity of tumor cells. Interestingly, after treatment with quemliclustat, tumors with a maximal decrease in *NR4A* expression exhibited a significant increase in multiple T cell activation signatures, upregulation of T cell lineage markers and markers of T cell activation. Additionally, the degree of tumor *NR4A* expression downregulation after treatment in 37 paired pretreatment/posttreatment tumor biopsies was associated with a significant improvement in OS (hazard ratio = 0.24 (95% CI: 0.08–0.67); *P* = 0.0035). These findings potentially suggest that a major driver behind the clinical benefit in ARC-8 may have been facilitated by quemliclustat-mediated modulation of an immunosuppressive, adenosine-rich TME and a subsequent increase in the abundance and activation of cytotoxic T cells in the vicinity of cancer cells.

As a phase 1b trial, the ARC-8 study was designed to evaluate the safety and tolerability of quemliclustat combined with G/nP with or without zimberelimab. The findings from ARC-8 may not be generalizable to the broader patient population, despite the sample size being large for an early phase trial. There was no concurrent, randomized control group. The post hoc SCA analysis allowed for exploratory comparisons with relevant historical clinical trial data that were propensity score matched to the ARC-8 Quemli100 cohort.

The promising OS profile observed in ARC-8, together with our mechanistic observations that quemliclustat treatment leads to a reduction in adenosine-regulated *NR4A* expression and subsequent T cell activation in the TME, supports further development of quemliclustat in patients with mPDAC. The ongoing phase 3 PRISM-1 trial (NCT06608927) is investigating quemliclustat and chemotherapy versus placebo and chemotherapy in patients with treatment-naive mPDAC.

## Methods

### Patients

Complete eligibility criteria are shown in Supplementary Table 6. Sex was recorded as a binary variable based on self-reported biological characteristics. Gender identity was not collected or analyzed in this study. No analyses stratified by sex were conducted.

### Study design

The study design for the ARC-8 trial is provided in Fig. 1. The primary endpoints included safety and tolerability of quemliclustat combination therapy. Secondary endpoints included ORR, DCR, DOR, PFS and OS. Additional planned secondary endpoints not reported in this paper are plasma concentration and pharmacokinetic parameters for quemliclustat, serum concentration and pharmacokinetic parameters for zimberelimab and number and percentage of patients who develop antidrug antibodies to zimberelimab.

The study was conducted in full conformance with the Declaration of Helsinki, the Council for International Organizations of Medical Sciences International Ethical Guidelines, institutional review board regulations and all other applicable local regulations. The study protocol was approved by the local ethics committee at each site (Supplementary Table 7). All patients provided written informed consent before any study procedures, and patients were not compensated monetarily for participation in this trial.

#### Dose-escalation phase

The dose-escalation phase employed a ‘3 + 3’ design with a 28-day DLT evaluation period (Supplementary Table 8). Three patients were enrolled in the initial dose cohort. Patients were considered evaluable for DLT if they received at least one dose of quemliclustat, received at least one dose of zimberelimab and completed the 28-day DLT evaluation period or experienced a DLT during the DLT evaluation period. When a minimum of three DLT-evaluable patients completed the DLT evaluation period for a given quemliclustat dose level, subsequent patients could be enrolled at the same, a lower or a higher dose level, and up to six patients could be treated at each dose level. The planned sample size for the dose-escalation phase was approximately 30 patients, based on the ‘3 + 3’ design. A patient not DLT evaluable was replaced with another patient at the same dose level. Disease status was evaluated every 8 weeks until disease progression (regardless of whether the patient was still receiving study treatment), study discontinuation or initiation of an alternative anticancer treatment.

Patients received quemliclustat intravenously (25 mg, 50 mg, 75 mg, 100 mg or 125 mg) every 2 weeks, G/nP (gemcitabine 1,000 mg m^−^^2^ and nab-paclitaxel 125 mg m^−^^2^) intravenously on days 1, 8 and 15 of a 28-day cycle and zimberelimab 240 mg intravenously every 2 weeks. The maximum tolerated dose was defined as the maximum dose at which fewer than 33% of patients experienced a DLT. The RP2D of quemliclustat was selected based on overall safety and tolerability, pharmacokinetics and pharmacodynamics.

#### Dose-expansion phase

The dose-expansion phase evaluated the RP2D of quemliclustat in patients with treatment-naive mPDAC. Initially, a single non-randomized arm of patients received the quemliclustat RP2D combined with G/nP and zimberelimab. After the prespecified interim analysis of the non-randomized arm, two additional arms were opened, and patients were enrolled and randomized 2:1 using the permuted block method to receive the quemliclustat RP2D combined with G/nP with or without zimberelimab. Subsequent prespecified data reviews were conducted every 6 months or after 45 patients were randomized and disease evaluable across both randomized arms, whichever occurred first.

ARC-8 (NCT04104672) was registered on 24 September 2019 with ClinicalTrials.gov.

### Assessments

Tumor response was assessed by investigators using Response Evaluation Criteria in Solid Tumors v.1.1. Endpoints included ORR based on confirmed and unconfirmed best overall response, PFS and OS.

Safety data included type, incidence, seriousness, causality and severity of TEAEs and serious adverse events, as assessed by investigators according to the National Cancer Institute’s Common Terminology Criteria for Adverse Events v.5.0 (ref. ^47^). Adverse events were coded using the Medical Dictionary for Regulatory Activities v.26.1.

### Statistical analysis

Analyses were based on the safety-evaluable population, defined as all patients who received at least one dose of any study treatment. The sample size justification was based on an estimation framework, and the study was designed for descriptive statistical analysis rather than formal statistical hypothesis testing involving power and type I error considerations. The planned sample size for the randomization portion of the dose-expansion phase was approximately 90 patients in a 2:1 ratio, with approximately 60 patients in the Q + G/nP + Z arm and approximately 30 patients in the Q + G/nP arm. Assuming a 40% ORR (24/60) in the Q + G/nP + Z arm and a 20% ORR (6/30) in the Q + G/nP arm, the 90% CI for the difference in ORR would be 3–37%, with the lower bound excluding the null value of 0%.

ORR was defined as the percentage of patients with a best overall response of complete or partial response and summarized with two-sided 95% CIs using the Clopper−Pearson method^48^. DCR was defined as the percentage of patients with a best overall response of complete response, partial response or stable disease. DOR was defined as the time from first documentation of disease response (complete or partial response) until first documentation of progressive disease or death, whichever occurs first. Responders without documented disease progression who were still alive at the time of analysis were censored at the time of their last tumor assessment. Median DOR was estimated using the Kaplan−Meier method, with two-sided 95% CIs calculated using the Brookmeyer−Crowley method. PFS was defined as the time from first dose to progressive disease or death due to any cause. OS was defined as the time from the first dose of study drug to death due to any cause. OS and PFS were estimated using Kaplan−Meier methodology. Median time to event for OS and PFS with two-sided 95% CIs was estimated using the Brookmeyer−Crowley method.

This interim analysis assessed emerging efficacy and safety data after all patients had been followed for at least 18 months. The analysis was not prespecified in the protocol and did not include formal futility boundaries; therefore, it was descriptive in nature and not intended to support definitive conclusions regarding efficacy or futility. Datasets for the clinical trial were prepared using standards from Clinical Data Interchange Consortium Study Data Tabulation Model implementation for human clinical trials and the Analysis Dataset Model.

### SCA analysis

In a post hoc analysis, we constructed an SCA to evaluate the efficacy of quemliclustat in patients with mPDAC, given that recruiting a control group for this patient population is challenging. The SCA analysis compared the efficacy outcomes of ARC-8 to the outcomes of a cohort of patients treated with G/nP alone in historical clinical trials. Patients with first-line mPDAC who received at least one dose of quemliclustat 100 mg in combination with G/nP with or without zimberelimab in the dose-escalation or dose-expansion phases of ARC-8 were compared to similar patients from historical clinical trials in first-line mPDAC who were treated with G/nP alone.

Historical clinical trial patient-level data for the SCA were sourced from electronic data capture available through an optional data-sharing program (Medidata Solutions, Inc., a Dassault Systèmes company). All available interventional trials completed before 7 June 2023 that enrolled adults with first-line mPDAC with a design that provided an opportunity to be assigned to G/nP were included in the search for eligible SCA patients. Up to four completed historical phase 2 or phase 3 randomized clinical trials were identified. All patients within these trials who satisfied prespecified SCA eligibility criteria (Supplementary Table 3), which were patterned after key eligibility criteria of the ARC-8 study, were included in the SCA-eligible cohort. Trials and patients were selected while blinded to all patient-level outcomes. Patient-level data, including baseline, outcome, prognostic and other variable definitions and conventions, were aligned to create a harmonized analysis dataset across historical clinical trials and ARC-8 using the data specifications from the ARC-8 study.

Propensity score methods commonly used to analyze observational data to reduce bias due to confounding variables that are unbalanced between groups of interest were used to create a one-to-one matching ratio between the Quemli100 cohort in ARC-8 and the SCA^49,50^. Specifically, greedy nearest neighbor propensity score matching without replacement, no caliper restriction and exact matching on the presence of liver metastases at baseline were used. All other available baseline and clinically important covariates considered necessary to achieve a well-balanced comparator group were included in the propensity score model (Supplementary Table 3). Balance was assessed using absolute standardized difference in covariate means^51,52^. Absolute standardized differences less than 0.25 were defined in a prespecified statistical analysis plan as indicating sufficiently well-balanced groups, with values less than 0.10 indicating negligible differences^53,54^. All propensity score modeling was completed while blinded to patient-level outcome data.

Efficacy endpoints of interest included OS, PFS and unconfirmed ORR. The index date for calculating OS and PFS was the date of first dose of study medication. Treatment effects for OS, PFS and unconfirmed ORR were analyzed by comparing the Quemli100 cohort in ARC-8 with the SCA. The Kaplan−Meier method was used to estimate PFS and OS rates at specified time intervals. Data for the SCA are part of the Medidata data-sharing program and were collected using Rave Electronic Data Capture. The data were extracted and standardized to ADaM datasets in SAS v.9.4.

### Cell culture experiments

Cell lines were purchased from the American Type Culture Collection and cultured based on the supplier’s recommendations. CAFs from a human pancreatic tumor were purchased from Neuromics (no. CAF118) and cultured based on the supplier’s recommendations. Human T cells were isolated from healthy donor blood using EasySep Human T Cell Isolation Kits (STEMCELL Technologies, 17592 and 17953). Cells were incubated in the presence of AMP (Thermo Fisher Scientific, J61643.06) and EHNA (Sigma-Aldrich, 324630) or NECA (Sigma-Aldrich, E2387) for 6 hours before RNA extraction. Total RNA was extracted using the RNeasy Mini Kit (Qiagen) according to the manufacturer’s instructions. cDNA was synthesized using SuperScript IV First-Strand Synthesis System (Thermo Fisher Scientific, 18-091-050), and real-time PCR was carried out using TaqMan (Thermo Fisher Scientific) assays *NR4A1* (Hs00374226_m1; cat. no. 4331182), *NR4A2* (Hs01117527_g1; cat. no. 4331182), *NR4A3* (Hs00545009_g1; cat. no. 4331182), HPRT1 (Hs02800695_m1; cat. no. 4448489) and ACTB (Hs01060665_g1; cat. no. 4448484). For RNA-seq, libraries were prepared using Illumina Stranded mRNA Prep. Sequencing was performed using Illumina NovaSeq 6000 at 150-bp paired-end reads for 20 million paired-end (40 million total) reads per sample.

### Tumor sample analysis

Pretreatment tumor biopsy was mandatory, whereas on-treatment biopsy was optional. Tumor formalin-fixed paraffin-embedded (FFPE) samples were profiled using RNA-seq. RNA was extracted from FFPE samples using the MagMAX FFPE DNA/RNA Ultra Kit (Thermo Fisher Scientific). Macrodissection was performed to enrich for 70% tumor content where possible. RNA-seq libraries were prepared with the TruSeq RNA Exome Kit (Illumina). Sequencing was performed on Illumina systems using 150-bp paired-end, dual-index reads. Samples were sequenced to a depth of 100 million paired-end (200 million total) reads.

### RNA-seq preprocessing

Quality control was performed using FASTQC^55^. Reads were aligned to the GRCh38 human reference genome (Ensembl v.104) using the STAR aligner^56^, and quantification was performed using Salmon^57^ with GENCODE v.38 annotations^58^. Count data were further normalized using library size adjustment and trimmed mean of *M*-values normalization, followed by voom transformation^59^.

### Differential gene expression analysis

Limma-voom^59^ with precision weights was used for differential gene expression analysis comparing different experimental conditions as contrasts using the following formula for the model matrix, where ~ indicates ‘modeled as/by’:

$$\sim \,0+\mathrm{Treatment}$$

For CD8 T cells, the model is adjusted for donor, as follows:

$$\sim \,0+\mathrm{Treatment}+\mathrm{Donor}$$

Genes were considered significantly upregulated by AMP + EHNA if they showed fold change ≥ 50% and adjusted *P* < 0.05 across all tested cell types. For quemliclustat inhibition analysis in cancer cell lines, genes were considered significantly inhibited if they showed ≥50% reduction in expression when quemliclustat was co-treated with AMP + EHNA compared to AMP + EHNA alone, with adjusted *P* < 0.05.

Gene set enrichment analysis (GSEA) was performed on the ranking of *t*-statistics from the differential gene expression analyses using the Fast GSEA (FGSEA) package^60^. Gene set scores were calculated using single-sample GSEA (ssGSEA)^61^ implemented in the gene set variation analysis (GSVA) package^62^. ssGSEA calculates enrichment scores based on the cumulative distribution of gene expression ranks within each sample. The ssGSEA scores were calculated for consistency in comparisons among different studies.

### Analysis of snRNA-seq dataset

Processed snRNA-seq data were downloaded from the Gene Expression Omnibus (accession ID GSE202051 (ref. ^31^)) and were analyzed using the R programming language using the Seurat package. The author-normalized RNA assay and reductions for principal component analysis, Harmony and uniform manifold approximation and projection were extracted and converted to a Seurat object for further analysis. Author cell type annotations were used for analysis. AUCell was used to calculate gene set scores for the *NR4A* family^63^. We applied the PurIST algorithm, which may be used accurately on low-input and degraded samples^64^, to the RNA-seq data. We classified patients from the ARC-8 study as classical molecular subtype or basal-like molecular subtype.

### Dual ISH

Dual ISH was used to detect mRNA transcripts for *NR4A1* and *IFNγ* in FFPE tissue sections. Staining was performed on the Leica Bond Rx automated staining platform using the RNAscope 2.5 LS Duplex Reagent Kit (Advanced Cell Diagnostics, 322440) according to the manufacturer’s recommended protocol. In brief, FFPE tissue sections (4–5 µm) were air dried or baked at 60 °C for 30 minutes, and deparaffinization and rehydration were performed according to the standard Leica Bond protocol. Peroxidase blocking and pretreatment with Protease III were performed with the aforementioned kit according to the specified protocol for the automated red/green duplex assay. Hybridization was simultaneously performed with probe 1 (red channel, C1; LS 2.5 Probe-Hs-*IFNG*, RNascope, 310508) and probe 2 (green channel, C2; 2.5 LS Probe-Hs-*NR4A1*-C2, RNascope, 851028-C2) supplied at 50× and diluted to 1× in probe diluent (Advanced Cell Diagnostics). After probe hybridization, 10 rounds of amplification and thorough washing steps were performed, followed by green and red chromogen deposition (Leica Biosystems; BOND Polymer Refine Red Detection, DS9390, and Green Chromogen, DC9913) and counterstaining according to the manufacturer’s recommended protocol. Additional probes were used as positive and negative controls (negative controls: 2.5 LS Duplex Control Probes (PPIB-C1, Polr2A-C2)-Human (RNAscope, 320748), 2.5 LS Duplex Negative Control Probe (DapB-C1, DapB-C2) (RNAscope, 320758)). Slides were air dried and mounted using EcoMount (Biocare Medical, EM897L). Probes for *NR4A2* (2.5 LS Probe-Hs-*NR4A2*; RNAscope, 582628) and NR4A3 (2.5 LS Probe-Hs-*NR4A3*; RNAscope, 575018) were also evaluated.

Whole-slide scanning was performed at ×40 magnification on the Pannoramic MIDI II Digital Scanner from 3DHISTECH (Epredia).

### mIF staining

mIF staining was performed on the Leica Bond Rx automated staining platform using the Opal 6-plex Detection Kit (Akoya Biosciences, NEL871001KT) for sequential staining of each marker. Markers were optimized, validated and tested in multiple staining positions subjected to multiple rounds of heat-induced epitope retrieval at pH 9.0 (Leica Biosystems; BOND Epitope Retrieval Solution 2, AR9640) to determine sequence position and antibody stripping efficiency using single-plex chromogenic detection before incorporation into the mIF panel. The final panel used included LAG3 (Leica Biosystems; clone 12H6, RTU Predilute, cat. no. PA0300, lot no. 79548), TOX (Cell Signaling Technology; clone E613Q, 1:1,500 dilution (0.045 µg ml^−1^), cat. no. 73758S, lot no. 1), CD3 (Leica Biosystems; clone LN10, RTU Predilute, cat. no. PA0553, lot no. 82525), PanCK (Abcam; clone AE1/AE3 + 5D3, 1:500 dilution (2 µg ml^−1^), cat. no. ab86734, lot no. GR3253264-1), FoxP3 (Cell Signaling Technology; clone D2W8E, 1:200 dilution (0.775 µg ml^−1^), cat. no. 98377S, lot no. 8) and CD8 (Cell Signaling Technology; clone D8A8Y, 1:200 dilution (0.125 µg ml^−1^), cat. no. 85336S, lot no. 5) in corresponding positions 1−6. FFPE slides were cut at 4–5-µm thickness on a Leica rotary microtome and baked at 60 °C for 1 hour; deparaffinization, rehydration, peroxidase blocking and antigen retrieval were performed according to the standard Leica Bond immunohistochemistry protocol. Each marker was detected in the order listed above using MACH2 Universal HRP Polymer (Biocare Medical, M2U522L) followed by OPAL dyes 690, 520, 570, 480 and 620 and, finally, OPAL TSA DIG followed by OPAL 780. A heat-induced primary antibody stripping step of 20 minutes at 98 °C in pH 9.0 buffer was performed after detection of each of the first five primary antibodies. Lastly, spectral DAPI was applied, and slides were mounted using ProLong Diamond Antifade Mountant (Life Technologies, P36961). Whole-slide scanning was performed at ×20 magnification on the Akoya Biosciences PhenoImager HT 2.0 spectral imaging system following the manufacturer’s instructions. All primary antibodies were validated for specificity by the respective manufacturers. Validation in the mIF setting was performed by comparing immunofluorescent staining patterns with the patterns obtained by gold standard chromogenic immunohistochemistry on tissue sections known to express the antibody targets.

### Digital image analysis

Biomarker quantification for mIF images and RNAscope images was performed using HALO software (v.3.6, Indica Labs). Images were annotated to select tissue regions for analysis. Artifacts, folds, necrotic regions and normal tissue regions, such as normal liver and pancreatic tissue, were excluded from analysis. DenseNET V2 HALO AI classifier was used to train and segment tissues into Tumor (epithelial), Stromal and Glass categories for chromogenic and fluorescent images. Tumor tissue classification was selected based on PanCK staining for mIF images, whereas tumor classification from RNAscope images was categorized based on tissue architecture and nuclear morphology according to RNAscope and hematoxylin and eosin images. The classification and analysis algorithms were trained on random images, and the optimized algorithm was applied to all images to perform batch analysis. Images that needed custom threshold levels were analyzed with a custom algorithm.

### mIF analysis

HighPlex FL module v.4.2.14.32 was used for mIF quantification. DAPI nuclear stain was used for detection and segmentation of nuclei. A positivity threshold was set for each marker based on nuclear or cytoplasmic expression and staining intensity such that no false positive or false negative was being quantified. Phenotypes were added according to the marker panel used in the 6-plex mIF. Immune phenotypes were quantified according to epithelial (tumor) and stromal compartments. Object data were saved for each image to acquire the positivity for individual markers and the co-localization of markers for phenotype detection as well as the *x* and *y* coordinates of all cells for performing further spatial analysis. The following phenotypes were detected from panel 1: PanCK^+^ (cancer cells), CD3^+^ (T cells), CD3^+^CD8^+^ (cytotoxic T cells), CD3^+^LAG3^+^ and CD8^+^LAG3^+^ (exhausted T cells) and CD3^+^FoxP3^+^ (regulatory T cells).

### Dual ISH analysis

ISH module v4.2.11.14 was used for RNAscope analysis. Green chromogen signal was used for detection of *NR4A1* and red chromogen for detection of *IFNγ*. An exclusion stain was designated to exclude any artifacts. RNAscope Cell Scoring within the ISH module was used for scoring probe copies according to 1+, 2+, 3+ and 4+ scores based on minimum copies per cell of 1, 4, 10 and 16, respectively. Quantification of probes was performed according to tumor and stroma classification previously described. Cells with *NR4A1* with fewer than four copies were termed as NR4A1^low^, and cells with *NR4A1* with more than 16 copies were termed as NR4A1^high^. Cells with no *NR4A1* copies were termed as NR4A1^negative^. Cells that were positive for *IFNγ* irrespective of copy number were termed as IFNγ^+^.

### Image fusing

Chromogenic RNAscope images were deconvoluted and converted into pseudofluorescent images using the Deconvolution module (v.2.0.1) in HALO software (v.4.0, Indica Labs). The deconvoluted RNAscope image was then registered with its corresponding mIF image using serial stain registration. The registered deconvoluted RNAscope image and mIF image were fused using serial stain fusing to create a composite image of ISH + mIF. The fused images generated were used for demonstrating the levels and spatial proximity of immune phenotypes, such as CD3^+^CD8^+^ in relation to NR4A1^high^ or NR4A1^low^ copy cells and expression of *IFNγ*.

### Spatial analysis

Spatial plots were generated using object data to show spatial location of immune phenotypes (CD8^+^, CD3^+^CD8^+^, CD3^+^LAG3^+^, CD8^+^Lag3^+^ and CD3^+^FoxP3^+^) in relation to PanCK^+^ cells and *NR4A1* high or low copy cells. Nearest neighbor and proximity analyses were performed using the HALO Spatial Analysis module. Nearest neighbor analysis was performed on RNAscope images to quantify the distance between IFNγ^+^ cells and NR4A1^negative^, NR4A1^low^ copy and NR4A1^high^ copy cells. Proximity analysis was performed on RNAscope images to quantify the percentage of IFNγ^+^ cells in 10-μm intervals within 50 μm from NR4A1^low^ copy and NR4A1^high^ copy cells. For mIF staining, proximity analysis was performed to measure CD3^+^ T cells and T cell subsets in 10-μm intervals within 50 μm of PanCK^+^ cancer cells.

### Survival analysis for biomarkers

All molecular biomarker data were analyzed for associations with clinical outcomes using the R programming language. Gene expression and signature analyses were performed by dividing patients into high versus low groups using median cutoffs or optimized thresholds, as specified in the corresponding figures. Optimal thresholds were determined using the bestcut algorithm in the survminer R package^65^, which identifies thresholds that maximize statistical significance of separation between groups. The parameter min.prop = 0.25 was applied, ensuring that at least 25% of samples fell into one of the two groups and reducing the likelihood of outliers driving the result. Kaplan−Meier survival analyses were conducted, with log-rank *P* values calculated using the survival and survminer R packages. Visualization of differences between defined groups was performed using ggplot2 and base R plotting functions. Forest plots were generated for *NR4A* family signature and *NR4A* family genes (*NR4A1*, *NR4A2*and *NR4A3*) across different clinical trials to determine hazard ratio and CI of each biomarker in relation to each other. Hazard ratios were calculated using continuous Cox regression analysis between scaled biomarker expression levels and survival outcomes.

### Data from external studies

For the PRINCE trial, RNA-seq data were downloaded from the publicly available GitHub repository associated with the original publication^12^ (https://github.com/ParkerICI/prince-trial-data). We used the author-processed data without further reprocessing. Clinical outcomes (OS and PFS) were examined biomarkers following the same procedures described in the Survival analysis for biomarkers section.

MORPHEUS-PDAC^37^ data were obtained from Roche through a data-sharing agreement. RNA-seq data were processed using the same pipeline described in the RNA-seq preprocessing section for the ARC-8 cohort. Clinical outcomes (OS and PFS) were examined in biomarkers following the same procedures described in the Survival analysis for biomarkers section.

### Software

SAS v.9.4 was used for analyses. Nextflow v.21.04 pipeline running nf-core/rnaseq v.3.0 with FASTQC 0.11.9, STAR v.2.6.1d and Salmon v.1.4.0 were used for quality control, alignment and quantification of RNA-seq data. Seurat v.5 and AUCell v.1.30 were used for single-cell RNA-seq analyses. Statistical analyses for biomarker associations and visualizations were conducted in R v.4.5 using the survminer, survival and ggplot packages. Digital image analyses were performed using HALO software v.3.6, and statistical analysis was performed using GraphPad Prism v.10.6.0 (build 890).

### Reporting summary

Further information on research design is available in the Nature Portfolio Reporting Summary linked to this article.

## Online content

Any methods, additional references, Nature Portfolio reporting summaries, source data, extended data, supplementary information, acknowledgements, peer review information; details of author contributions and competing interests; and statements of data and code availability are available at 10.1038/s41591-026-04283-z.

## Supplementary information

Supplementary Information Supplementary Tables 1–8

Reporting Summary

## Data Availability

Arcus Biosciences is committed to sharing clinical trial data with external qualified scientific researchers in the interest of advancing public health. Arcus Biosciences will provide access to individual deidentified participant data and related study documents (protocols, statistical analysis plans and clinical study reports) upon request from qualified researchers and subject to certain criteria, conditions and exceptions. For information on the process or to submit a request, visit https://trials.arcusbio.com/our-transparency-policy.
