# Supplementary material for: Quemliclustat and chemotherapy with or without zimberelimab in metastatic pancreatic adenocarcinoma: a randomized phase 1 trial
Source: Nat Med. 2026 Mar 30;32(4):1267–77. doi: 10.1038/s41591-026-04283-z (PMC13099643; doi:10.1038/s41591-026-04283-z)
Supplement: Supplementary file 1 — Supplementary Tables 1–8 [file 41591_2026_4283_MOESM1_ESM.pdf]

nature medicine

Supplementary information https://doi.org/10.1038/s41591-026-04283-z

Quemliclustat and chemotherapy with or without zimberelimab in metastatic pancreatic adenocarcinoma: a randomized phase 1 trial

In the format provided by the authors and unedited

Supplementary Material

Supplementary Table 1 | Treatment-emergent adverse events occurring in ≥20% of patients in the dose-expansion phase

Supplementary Table 2 | OS in the randomized arms of the dose-expansion phase by presence of liver metastasis at baseline

Supplementary Table 3 | Baseline and clinically important covariates considered for inclusion in the propensity score model

Supplementary Table 4 | Baseline demographics and disease characteristics for the SCA analysis before and after matching

Supplementary Table 5 | Baseline demographic and clinical characteristics of the ARC-8 biomarker evaluable population

Supplementary Table 6 | Inclusion and exclusion criteria

Supplementary Table 7 | Ethics committee information

Supplementary Table 8 | Definition of DLT (dose-escalation phase)

Supplementary Table 1 | Treatment-emergent adverse events occurring in ≥20% of patients in the dose-expansion phase

	Randomized		Aggregated	
**Incidence, *n* (%)**	**Q+G/nP (*n* = 29)**	**Q+G/nP+Z (*n* = 61)**	**Pooled Q+G/nP+Z (*n* = 93)**	**Quemli100 (*n* = 122)**
Fatigue	21 (72)	44 (72)	66 (71)	87 (71)
Nausea	20 (69)	38 (62)	53 (57)	73 (60)
Anemia	20 (69)	31 (51)	49 (53)	69 (57)
Diarrhea	15 (52)	27 (44)	47 (51)	62 (51)
Alopecia	15 (52)	26 (43)	43 (46)	58 (48)
Decreased neutrophil count	17 (59)	24 (39)	40 (43)	57 (47)
Pyrexia	11 (38)	26 (43)	45 (48)	56 (46)
Peripheral edema	13 (45)	30 (49)	43 (46)	56 (46)
Decreased platelet count	15 (52)	16 (26)	35 (38)	50 (41)
Abdominal pain	10 (35)	23 (38)	36 (39)	46 (38)
Decreased appetite	8 (28)	19 (31)	38 (41)	46 (38)
Increased AST	11 (38)	19 (31)	31 (33)	42 (34)
Constipation	9 (31)	22 (36)	33 (36)	42 (34)
Decreased white blood cell count	14 (48)	15 (25)	27 (29)	41 (34)
Increased ALT	12 (41)	18 (30)	28 (30)	40 (33)
Vomiting	11 (38)	17 (28)	26 (28)	37 (30)
Maculo-papular rash	9 (31)	18 (30)	28 (30)	37 (30)
Peripheral sensory neuropathy	8 (28)	17 (28)	26 (28)	34 (28)
Hypokalemia	10 (35)	11 (18)	20 (22)	30 (25)
Hypoalbuminemia	7 (24)	14 (23)	21 (23)	28 (23)
Hyponatremia	9 (31)	12 (20)	19 (20)	28 (23)
Dyspnea	8 (28)	12 (20)	20 (22)	28 (23)
Headache	5 (17)	18 (30)	23 (25)	28 (23)
Increased blood ALP	8 (28)	8 (13)	19 (20)	27 (22)
Pruritus	6 (21)	15 (25)	21 (23)	27 (22)
Hypertension	9 (31)	9 (15)	14 (15)	23 (19)
Cough	10 (35)	6 (10)	13 (14)	23 (19)
Peripheral neuropathy	10 (35)	8 (13)	12 (13)	22 (18)

Back pain	6 (21)	11 (18)	15 (16)	21 (17)
Urinary tract infection	6 (21)	9 (15)	12 (13)	18 (15)
Increased blood LDH	7 (24)	5 (8)	11 (12)	18 (15)
Hypocalcemia	6 (21)	8 (13)	11 (12)	17 (14)
Abdominal distension	6 (21)	5 (8)	10 (11)	16 (13)
Pain in extremity	6 (21)	7 (12)	9 (10)	15 (12)
Stomatitis	7 (24)	3 (5)	8 (9)	15 (12)

Data cutoff date for the dose-expansion phase was June 19, 2023.
ALP, alkaline phosphatase; ALT, alanine aminotransferase; AST, aspartate aminotransferase; G/nP, gemcitabine/nab-paclitaxel; LDH, lactate dehydrogenase; Pooled Q+G/nP+Z, all patients treated with Q and G/nP with Z; Q, quemliclustat 100 mg; Quemli100, all patients treated with Q and G/nP with or without Z; Z, zimberelimab.

Supplementary Table 2 | OS in the randomized arms of the dose-expansion phase by presence of liver metastasis at baseline

	Randomized		Aggregated	
**Parameter**	**Q+G/nP (*n* = 29)**	**Q+G/nP+Z (*n* = 61)**	**Pooled Q+G/nP+Z (*n* = 93)**	**Quemli100 (*n* = 122)**
Without liver metastasis at baseline				
n	12	19	31	43
Events (%)	4 (33)	7 (37)	16 (52)	20 (47)
Median OS,[a] months (95% CI)	22.0 (17.9–NE)	21.2 (14.6–NE)	21.2 (13.9–25.4)	21.5 (17.9–25.4)
With liver metastasis at baseline				
n	17	42	62	79
Events (%)	11 (65)	26 (62)	40 (65)	51 (65)
Median OS,[a] months (95% CI)	12.1 (10.0–20.9)	12.2 (6.2–17.9)	11.1 (8.1–14.5)	12.1 (10.0–15.7)

[a]Based on Kaplan-Meier analysis.

G/nP, gemcitabine/nab-paclitaxel; NE, not estimable; OS, overall survival; Pooled Q+G/nP+Z, all patients treated with Q and G/nP with Z; Q, quemliclustat 100 mg; Quemli100, all patients treated with Q and G/nP with or without Z; Z, zimberelimab.

Supplementary Table 3 | Baseline and clinically important covariates considered for inclusion in the propensity score model

Baseline variable	Type of collected variable (values/unit)
Presence of liver metastases at baseline	Categorical (yes, no)
Time since diagnosis	Continuous (months)
Prior surgery for pancreatic cancer	Categorical (yes, no)
Age	Continuous (years)
Sex	Categorical (female, male)
Race	Categorical (White, Black or African American, Asian, Native Hawaiian or Other Pacific Islander, American Indian or Alaskan Native, other, not reported[a])
Ethnicity	Categorical (Non-Hispanic or -Latino, Hispanic or Latino, not reported[a])
ECOG PS	Categorical (0, 1)

[a]Includes missing values and those recorded as “not reported.”
ECOG PS, Eastern Cooperative Oncology Group performance status.

Supplementary Table 4 | Baseline demographics and disease characteristics for the SCA analysis before and after matching

	Before matching			After matching		
**Characteristic**	**Quemli100 (*n* = 122)**	**SCA eligible (*n* = 515)**	**Standardized difference**	**Quemli100 (*n* = 122)**	**SCA (*n* = 122)**	**Standardized difference**
Liver metastasis at baseline,[a] *n* (%)	79 (65)	415 (81)	−0.361	79 (65)	79 (65)	0.000
Age, years, mean (SD)	64.5 (8.9)	64.4 (8.4)	0.011	64.5 (8.9)	64.9 (8.7)	−0.046
Sex, *n* (%)			0.085			0.016
Male	64 (53)	292 (57)		64 (53)	65 (53)	
Female	58 (48)	223 (43)		58 (48)	57 (47)	
Race, *n* (%)			−0.086			0.067
White/not reported	104 (85)	454 (88)		104 (85)	101 (83)	
Other	18 (15)	61 (12)		18 (15)	21 (17)	
Ethnicity, *n* (%)			0.111			0.000
Non-Hispanic or Latino or not reported	117 (96)	481 (93)		117 (96)	117 (96)	
Hispanic or Latino	5 (4)	34 (7)		5 (4)	5 (4)	
ECOG PS, *n* (%)			−0.270			−0.068
0	42 (34)	245 (48)		42 (34)	46 (38)	
1	80 (66)	270 (52)		80 (66)	76 (62)	
Duration since diagnosis, months, mean (SD)	4.7 (10.0)	3.8 (10.8)	0.089	4.7 (10.0)	4.5 (11.4)	0.020
Prior surgery for pancreatic cancer, *n* (%)			0.248			0.044
Yes	21 (17)	46 (9)		21 (17)	19 (16)	
No	101 (83)	469 (91)		101 (83)	103 (84)	
Propensity score, mean (SD)	0.24 (0.108)	0.18 (0.086)	0.550	0.24 (0.108)	0.23 (0.100)	0.034

[a]Exact matching on the presence of liver metastases at baseline was specified in the propensity score model.
ECOG PS, Eastern Cooperative Oncology Group performance status; G/nP, gemcitabine/nab-paclitaxel; Q, quemliclustat 100 mg; Quemli100, all patients treated with Q and G/nP with or without Z; SCA, synthetic control arm; Z, zimberelimab.

Supplementary Table 5 | Baseline demographic and clinical characteristics of the ARC-8 biomarker evaluable population

Characteristic	Total Quemli-treated (*n* = 122)	Biomarker (RNA-seq) evaluable	
		Baseline (*n* = 80)	All paired biopsies (*n* = 37)
Age, years, median (range)	65.5 (41–81)	67.5 (42–81)	66 (44–81)
Sex, *n* (%)			
Female	58 (48)	37 (46)	20 (54)
Male	64 (53)	43 (54)	17 (46)
ECOG PS, 0/1, %	34/66	36/64	49/51
Liver metastases present at baseline, *n* (%)	79 (65)	56 (70)	26 (70)
Median overall survival, months (95% CI)	15.7 (12.4–20.9)	13.9 (10.8–21.5)	16.5 (11.3–25.4)

ECOG PS, Eastern Cooperative Oncology Group performance status; Quemli, quemliclustat with or without zimberelimab; RNA-seq, RNA sequencing.

Supplementary Table 6 | Inclusion and exclusion criteria

Patients are eligible to be included in the study only if all the criteria below apply

1. Capable of giving signed informed consent
2. Male or female patients aged ≥18 years at the time of screening
3. Women with no childbearing potential because of surgery or who are at least 1 year postmenopausal (ie, 12 months after the last menstrual period) or with menopause confirmed by follicle-stimulating hormone testing
4. Women of childbearing potential must use a highly effective method of contraception
5. A negative serum pregnancy test at screening and before dosing on cycle 1 day 1 and a negative serum or urine pregnancy test on the first day of each subsequent treatment period (women of childbearing potential only)
6. Males must use an effective method of contraception (condom, occlusive cap (diaphragm or cervical or vault caps) with spermicidal foam or gel or film or cream or suppository, or vasectomy)
7. Histologically or cytologically confirmed metastatic pancreatic adenocarcinoma
8. Must be naive to any prior treatment for metastatic disease, including chemotherapy, biological therapy, or targeted therapy
    a. Prior adjuvant therapy (including chemotherapy and/or radiotherapy) for pancreatic adenocarcinoma is permitted if neoadjuvant or adjuvant therapy was completed at least 6 months before study enrollment. Prior adjuvant therapy may include G/nP
    b. Patients initially diagnosed with locally advanced pancreatic cancer who have undergone chemotherapy then resection and had no evidence of disease are eligible if relapse of metastatic disease has occurred and if the last dose of chemotherapy was received more than 6 months before study entry
9. Must have at least one measurable lesion per RECIST v1.1. The measurable lesion must be outside of a radiation field if the patient received prior radiation
10. ECOG PS of 0 or 1
11. Confirm that an archival tissue sample is aged ≤12 months and shipped. If not, a new biopsy of a tumor lesion not previously irradiated (tumors progressing in a prior site of radiation may be considered after sponsor consultation) should be obtained. The biopsy must not put patients at undue risk, and the procedure must not be more invasive than a core biopsy, as documented in the medical record by the investigator
12. Prior radiation therapy for metastatic disease must have been completed as follows:
    a. Prior systemic radiation or whole brain radiation at least 4 weeks before study treatment administration
    b. Prior focal radiotherapy at least 2 weeks before study treatment administration
    c. No radiopharmaceuticals (eg, strontium, samarium) may have been administered
13. Immunosuppressive doses of systemic medications, such as corticosteroids or absorbed topical corticosteroids (doses >10 mg/day prednisone or equivalent), must be discontinued at least 2 weeks (14 days) before study treatment administration. Physiologic

doses of corticosteroids (≤10 mg/day of prednisone or its equivalent) or short pulses of corticosteroids (≤3 days) may be permitted

14. Prior surgery that required general anesthesia or other major surgery, as defined by the investigator, must be completed at least 4 weeks before study treatment administration. Surgery requiring regional/epidural anesthesia must be completed at least 72 hours before study treatment administration. Patients should have recovered from the surgical procedure before the first dose being administered. Cutaneous biopsies with only local anesthesia should be completed at least 1 hour before study treatment administration
15. Patients must not have a history of HIV (HIV-1 or HIV-2), HBV, or HCV, except for the following:
    a. Patients with anti-hepatitis B core antibody but with undetectable HBV DNA and negative for hepatitis B surface antigen
    b. Patients with resolved or treated HCV (ie, HCV antibody positive but undetectable HCV RNA)
16. Adequate organ and marrow function, as defined below:
    a. Neutrophils ≥1000/μL (in the absence of growth factor support)
    b. Platelets ≥100 x 10^3/μL
    c. Hemoglobin ≥9.0 g/dL
    d. Serum creatinine ≤1.5 x ULN
    e. Albumin >2.8 g/dL
    f. AST ≤2.5 x ULN without hepatic metastasis and ≤5 x ULN with hepatic metastasis
    g. ALT ≤2.5 x ULN without hepatic metastasis and ≤5 x ULN with hepatic metastasis
    h. Total bilirubin ≤1.5 x ULN (except patients with Gilbert's syndrome who must have total bilirubin ≤5.0 mg/dL)

Patients are excluded from the study if any of the criteria below apply

1. Use of any live vaccines against infectious diseases (eg, influenza, varicella) within 4 weeks (28 days) of initiation of study treatment
2. Underlying medical conditions (eg, interstitial lung disease, active infections requiring antibiotics, recent hospitalization with unresolved symptoms, interstitial lung disease, active coagulopathy, ascites) that, in the investigator's or sponsor's opinion, will make the administration of study treatment hazardous or obscure the interpretation of toxicity determination or AEs
3. Significant cardiovascular disease (NYHA Class III–IV), myocardial infarction or cerebrovascular accident within 12 months of the first dose of investigational agent or history of arterial thromboembolic event, uncontrolled hypertension, unstable arrhythmia, or unstable angina within 3 months or venous thromboses within 1 month of the first dose of investigational agent. Patients with existing venous thromboses must be stable beyond a month, with no evidence of RV strain and no new thromboses since the time of diagnosis. Medical monitor approval is also required
4. Has known psychiatric or substance abuse disorders that would interfere with cooperation with the requirements of the trial
5. Is pregnant or breastfeeding or expecting to conceive or father children within the projected duration of the study, starting with the pre-screening or screening visit until up to 6 months after the last dose of quemliclustat combination therapy

6. Any active or documented history of autoimmune disease, including but not limited to inflammatory bowel disease, celiac disease, Wegner syndrome, Hashimoto syndrome, systemic lupus erythematosus, scleroderma, sarcoidosis, or autoimmune hepatitis, within 3 years of the first dose of study treatment, except for the following:
    a. Type I diabetes mellitus, hypothyroidism only requiring hormone replacement, skin disorders such as vitiligo, or alopecia not requiring systemic therapy, or conditions not expected to recur in the absence of an external trigger
    b. Endocrinopathies where the patient is stable on hormone replacement therapy
    c. History of Hashimoto syndrome within 3 years of the first study treatment that resolved to hypothyroidism alone
7. History of a syndrome that required systemic steroids or immunosuppressive medications, except for vitiligo or resolved childhood asthma/atopy. Patients with asthma who require intermittent use of bronchodilators (such as albuterol) will not be excluded from this study.
8. Any acute gastrointestinal symptoms (eg, nausea, vomiting, diarrhea) at the time of screening or admission.
9. Prior active malignancy within the previous year except for locally curable cancers that have been apparently cured, such as basal or squamous cell skin cancer, superficial bladder cancer, or carcinoma *in situ* of the cervix, breast, or prostate cancer.
10. Has not recovered from a non-hematologic AEs due to a previously administered agent, except grade 2 alopecia or grade 2 neuropathy. However, if the AE is deemed stable in the opinion of the investigator, the patient may be considered eligible after discussion with the medical monitor.

AE, adverse event; ALT, alanine aminotransferase; AST, aspartate aminotransferase; ECOG PS, Eastern Cooperative Oncology Group performance status; HBV, hepatitis B virus; HCV, hepatitis C virus; NYHA, New York Heart Association; RV, right ventricular; ULN, upper limit of normal.

Supplementary Table 7 | Ethics committee information

Centre Number	Institution Name	IRB name
30201	Sarah Cannon Research Institute	Advarra
30202	University of Pittsburgh Medical Center Hilman Cancer Center	WCG
30203	University of California, Los Angeles, Hematology Oncology	UCLA IRB
30204	Yale Cancer Center	WCG
30205	New York University Langone Medical Center - New York University Medical Oncology Associates	New York University Langone Health IRB
30206	The University of Texas MD Anderson Cancer Center	The University of Texas MD Anderson Cancer Center IRB
30207	Columbia University Medical Center, New York Presbyterian Hospital	Columbia University IRB
30208	Memorial Sloan Kettering Cancer Center - Main Campus	Memorial Sloan Kettering Cancer Center IRB
30209	Thomas Jefferson, University, Sidney Kimmel Cancer Center	Advarra
30210	Washington University School of Medicine - Siteman Cancer Center	WCG
30211	University of Oklahoma Health Sciences Center-Peggy and Charles Stephenson Oklahoma Cancer Center	Advarra
30212	University of Pennsylvania Health System	WCG
30213	University of Wisconsin Hospitals and Clinics	WCG
30214	The Angeles Clinic and Research Institute	WCG & Cedars-Sinai's IRB
30216	Medical Oncology Associates, Ps, Summit Cancer Centers	WCG
30217	Mid-Florida Hematology & Oncology Centers, PA	WCG
30218	START - South Texas Accelerated Research Therapeutics, LLC	Salus IRB
30219	BRCR Global	WCG

IRB, Institutional Review Board; WCG, WIRB-Copernicus Group, Inc.

Supplementary Table 8 | Definition of DLT (dose-escalation phase)

The occurrence of any of the following toxicities was considered a DLT if judged by the investigator to be possibly, probably, or definitely related to experimental therapy

1. Grade 4 non-hematologic toxicity (not laboratory)
2. Grade 3 non-hematologic, non-laboratory toxicity, with the exception of nausea, vomiting and diarrhea, that resolves to grade ≤1 within 3 days using optimal supportive care
3. Any grade 3 or 4 clinically significant non-hematologic laboratory value if:
    a. The abnormality leads to hospitalization, or
    b. The abnormality persists despite optimal supportive care/repletion
4. Grade 3 or 4 febrile neutropenia
5. Grade 3 thrombocytopenia with clinically significant bleeding (not limited to requiring transfusion) and grade 4 thrombocytopenia (regardless of bleeding)
6. Prolonged delay (>4 weeks) in initiating cycle 2 of G/nP due to treatment-related toxicity
7. Grade 5 toxicity

Any toxicity that did not meet the above defined criteria for a DLT but was judged by the investigator to be possibly, probably, or definitely related to experimental therapy and resulted in a dose delay, hold, or reduction was considered a DLT after discussion and agreement with the medical monitor. The DLT evaluation period was defined as the first 28 days of dosing

DLT, dose-limiting toxicity; G/nP, gemcitabine/nap-paclitaxel.
