# Supplementary material for: Quemliclustat and chemotherapy with or without zimberelimab in metastatic pancreatic adenocarcinoma: a randomized phase 1 trial
Source: Nat Med. 2026 Mar 30;32(4):1267–77. doi: 10.1038/s41591-026-04283-z (PMC13099643; doi:10.1038/s41591-026-04283-z)
Supplement: Supplementary file 2 — Reporting Summary [file 41591_2026_4283_MOESM2_ESM.pdf]

nature portfolio

Corresponding author(s): Zev A Wainberg, MD
Eileen M O'Reilly, MD, FASCO
Omar Kabbarah, PhD

Last updated by author(s): Jan 9, 2026

Reporting Summary

Nature Portfolio wishes to improve the reproducibility of the work that we publish. This form provides structure for consistency and transparency in reporting. For further information on Nature Portfolio policies, see our Editorial Policies and the Editorial Policy Checklist.

Statistics

For all statistical analyses, confirm that the following items are present in the figure legend, table legend, main text, or Methods section.

n/a	Confirmed	
☐	☒	The exact sample size (*n*) for each experimental group/condition, given as a discrete number and unit of measurement
☐	☒	A statement on whether measurements were taken from distinct samples or whether the same sample was measured repeatedly
☐	☒	The statistical test(s) used AND whether they are one- or two-sided<br>*Only common tests should be described solely by name; describe more complex techniques in the Methods section.*
☐	☒	A description of all covariates tested
☐	☒	A description of any assumptions or corrections, such as tests of normality and adjustment for multiple comparisons
☐	☒	A full description of the statistical parameters including central tendency (e.g. means) or other basic estimates (e.g. regression coefficient) AND variation (e.g. standard deviation) or associated estimates of uncertainty (e.g. confidence intervals)
☐	☒	For null hypothesis testing, the test statistic (e.g. *F*, *t*, *r*) with confidence intervals, effect sizes, degrees of freedom and *P* value noted<br>*Give P values as exact values whenever suitable.*
☒	☐	For Bayesian analysis, information on the choice of priors and Markov chain Monte Carlo settings
☒	☐	For hierarchical and complex designs, identification of the appropriate level for tests and full reporting of outcomes
☒	☐	Estimates of effect sizes (e.g. Cohen's *d*, Pearson's *r*), indicating how they were calculated

Our web collection on statistics for biologists contains articles on many of the points above.

Software and code

Policy information about availability of computer code

Data collection	Datasets for the clinical trial were prepared using standards from Clinical Data Interchange Consortium Study Data Tabulation Model implementation for human clinical trials and Analysis Dataset Model.<br>Data for the synthetic control arm is part of the Medidata data-sharing program and was collected using Rave Electronic Data Capture. The data were extracted and standardized to ADaM datasets in SAS v9.4.<br>RNAseq data from PRINCE trial obtained from "https://github.com/ParkerICI/prince-trial-data" and Gene Expression Omnibus, GSE202051.<br>Dual ISH images were collected at 40X magnification on the Pannoramic MIDI II digital scanner and whole-slide scanning for mIF was performed at 20X magnification on the Akoya Biosciences PhenoImager.
Data analysis	SAS v9.4 was used for analyses. Nextflow v.21.04 pipeline running nf-core/rnaseq v3.0 with FASTQC 0.11.9, STAR v2.6.1d, and salmon v1.4.0 were used for quality control, alignment, and quantification of RNAseq data. Seurat v5 and AUCell v1.30 were used for scRNAseq analyses. Statisical analyses for biomarker associations and visualizations were conducted in R v4.5 using survminer, survival, and ggplot packages. Parameters used for each software are described in the methods. Digital image analyses were performed using HALO software and statistical analysis was performed using Graph pad PRISM v10.6.0 (890). HALO modules used for specific analysis are described in the methods.

For manuscripts utilizing custom algorithms or software that are central to the research but not yet described in published literature, software must be made available to editors and reviewers. We strongly encourage code deposition in a community repository (e.g. GitHub). See the Nature Portfolio guidelines for submitting code & software for further information.

Data

Policy information about availability of data

All manuscripts must include a data availability statement. This statement should provide the following information, where applicable:

- Accession codes, unique identifiers, or web links for publicly available datasets
- A description of any restrictions on data availability
- For clinical datasets or third party data, please ensure that the statement adheres to our policy

Arcus Biosciences is committed to responsible sharing of data from clinical trials we sponsor. This includes summary and de-identified individual patient data, as well as other trial information (protocols, statistical analysis plans and clinical study reports). Requests for data from any qualified researcher who engages in rigorous, independent scientific research will be considered if the clinical trial data are not part of an ongoing or planned regulatory submission. Original data will be available for 12 months, beginning 3 months after approval of the study drug for use in patients or a new indication. For information on the process or to submit a request, visit https://trials.arcusbio.com/our-transparency-policy.

Datasets for the clinical trial were prepared using standards from Clinical Data Interchange Consortium Study Data Tabulation Model implementation for human clinical trials and Analysis Dataset Model.
Data for the synthetic control arm is part of the Medidata data-sharing program and was collected using Rave Electronic Data Capture. The data were extracted and standardized to ADaM datasets in SAS v9.4.
RNAseq data from PRINCE trial obtained from "https://github.com/ParkerICI/prince-trial-data" and Gene Expression Omnibus, GSE202051. Dual ISH images were collected at 40X magnification on the Pannoramic MIDI II digital scanner and whole-slide scanning for mIF was performed at 20X magnification on the Akoya Biosciences PhenoImager.

SAS v9.4 was used for analyses. Nextflow v.21.04 pipeline running nf-core/rnaseq v3.0 with FASTQC 0.11.9, STAR v2.6.1d, and salmon v1.4.0 were used for quality control, alignment, and quantification of RNAseq data. Seurat v5 and AUCell v1.30 were used for scRNAseq analyses. Statisical analyses for biomarker associations and visualizations were conducted in R v4.5 using survminer, survival, and ggplot packages. Parameters used for each software are described in the methods. Digital image analyses were performed using HALO software v3.6 and statistical analysis was performed using Graph pad PRISM. HALO modules used for specific analysis are described in the methods.

Research involving human participants, their data, or biological material

Policy information about studies with human participants or human data. See also policy information about sex, gender (identity/presentation), and sexual orientation and race, ethnicity and racism.

Reporting on sex and gender	Sex was recorded as a binary variable based on self-reported biological characteristics. Gender identity was not collected or analyzed in this study. No analyses stratified by sex were conducted. Table 2 and Extended Data Table 1 report breakdown by sex (female and male) of the patients.
Reporting on race, ethnicity, or other socially relevant groupings	Race and/or ethnicity were determined and classified based on self-report.
Population characteristics	The ARC-8 study was conducted at 18 clinical sites in the United States. Patients were aged ≥ 18 years with a histologically or cytologically confirmed diagnosis of mPDAC, had no previous treatment for metastatic disease and had ECOG performance status of 0 or 1. Complete eligibility criteria are shown in Extended Data Table 6. Demographic and baseline disease characteristics are shown in Table 2 and Extended Data Table 1.
Recruitment	The ARC-8 study was recruited at 18 clinical sites in the United States. Participants were recruited by investigators at each participating study site. Recruiting bias is not expected to be higher on this study than on other clinical trials of similar phase and size, and is not anticipated to substantially impact results.
Ethics oversight	No central IRB or ethics committee was used. At each site, the study was conducted in adherence to the requirements of 21 Code of Federal Regulations, International Council for Harmonization guidelines, institutional review board regulations and all other applicable local regulations. All patients provided written informed consent. This study was conducted in full conformance with the International Council for Harmonization E6 guideline for Good Clinical Practice and the consensus ethical principles derived from international guidelines, including the Declaration of Helsinki and Council for International Organizations of Medical Sciences International Ethical Guidelines, and applicable laws and regulations. The study was conducted in the United States under a US Investigational New Drug application and complied with US Food and Drug Administration regulations, including all applicable local, state and federal laws.<br><br>The protocol was approved by the local ethics committee at each site (Supplementary Table 7). All patients provided written informed consent; patients were not compensated monetarily for their participation in this trial.

Note that full information on the approval of the study protocol must also be provided in the manuscript.

Field-specific reporting

Please select the one below that is the best fit for your research. If you are not sure, read the appropriate sections before making your selection.

☒ Life sciences ☐ Behavioural & social sciences ☐ Ecological, evolutionary & environmental sciences

Life sciences study design

All studies must disclose on these points even when the disclosure is negative.

Sample size
The planned sample size for dose escalation was approximately 30 participants, depending on the toxicities observed.
The dose-expansion portion of the trial is intended to further characterize safety and potential anti-tumor activity, and to begin assessing potential treatment benefit via open-label randomization. As such, the sample size justification was largely based on an estimation framework rather than formal Type I error and power considerations. The decision to open randomization for the dose expansion was based on an interim analysis of the non-randomized arm, conducted after the non-randomized arm enrolled at least 15 participants who were evaluable for disease assessment and reviewed by an independent monitoring committee. The planned sample size for the randomized portion of the dose expansion was approximately 90 patients in a 2:1 ratio, with approximately 60 patients in the Q+G/nP+Z arm and approximately 30 patients in the Q+G/nP arm. Of the 122 patients aggregated for the Quemli100 cohort, 80 were evaluable by RNAseq.

Data exclusions
Inclusion/exclusion criteria are described in the methods and in Extended Data Table 6. The goal of the study was to assess the safety and tolerability of quemliclustat combination therapy in participants with advanced gastrointestinal malignancies.

Replication
This was a clinical trial. No replication was performed.

Randomization
All participants were centrally assigned to study treatment using an Interactive Voice/Web Response System. Directions and log in/contact information for the Interactive Voice/Web Response System were provided to each site. In the randomized arm of the dose expansion phase, patients were randomized to treatment groups in a 2:1 ratio using a permuted block method.

Blinding
This is an open-label trial; therefore, the sponsor, investigator, and participant know the study treatment administered.

Reporting for specific materials, systems and methods

We require information from authors about some types of materials, experimental systems and methods used in many studies. Here, indicate whether each material, system or method listed is relevant to your study. If you are not sure if a list item applies to your research, read the appropriate section before selecting a response.

Materials & experimental systems

n/a	Involved in the study	
☐	☒	Antibodies
☐	☒	Eukaryotic cell lines
☒	☐	Palaeontology and archaeology
☒	☐	Animals and other organisms
☐	☒	Clinical data
☒	☐	Dual use research of concern
☒	☐	Plants

Methods

n/a	Involved in the study	
☒	☐	ChIP-seq
☒	☐	Flow cytometry
☒	☐	MRI-based neuroimaging

Antibodies

Antibodies used
LAG3 (clone 12H6, RTU Predilute, Cat# PA0300, Lot# 79548, Leica Biosystems, Deer Park, Illinois), TOX (clone E613Q, 1:1500 dilution [0.045 ug/ml], Cat# 73758S, Lot# 1, Cell Signaling Technology, Danvers, Massachusetts), CD3 (clone LN10, RTU Predilute, Cat# PA0553, Lot# 82525, Leica Biosystems, Deer Park, Illinois), PanCK (clone AE1/AE3 + 5D3, 1:500 dilution [2ug/ml], Cat# ab86734, Lot# GR3253264-1, Cambridge, United Kingdom), FoxP3 (clone D2W8E, 1:200 dilution [0.775 ug/ml], Cat# 98377S, Lot# 8, Cell Signaling Technology, Danvers, Massachusetts) and CD8 (clone D8A8Y, 1:200 dilution [0.125 ug/ml], Cat# 85336S, Lot# 5, Cell Signaling Technology, Danvers, Massachusetts).

Validation
All primary antibodies were validated for specificity by the respective manufacturers. Validation in the mIF setting was performed by comparing immunofluorescent staining patterns to the patterns obtained by gold-standard chromogenic immunohistochemistry on tissue sections known to express the antibody targets.

Eukaryotic cell lines

Policy information about cell lines and Sex and Gender in Research

Cell line source(s)
PANC-1 (ATCC_CRL-1469), MIA Paca-2 (ATCC_CRL-1420), Pancreatic CAF cells (Neuromics CAF118)

Authentication
Cell lines were validated by the manufacturers with corresponding certificate of analysis

Mycoplasma contamination
All cell lines were tested negative for Mycoplasma

Commonly misidentified lines (See ICLAC register)	No commonly misidentified lines were used in this study

Clinical data

Policy information about clinical studies

All manuscripts should comply with the ICMJE guidelines for publication of clinical research and a completed CONSORT checklist must be included with all submissions.

Clinical trial registration	NCT04104672
Study protocol	The redacted protocol and the statistical analysis plan are provided in the supplemental materials.
Data collection	The dose escalation portion enrolled patients starting on February 3, 2020 (first patient, first visit) and 3 patients are ongoing. The dose escalation data cutoff date was February 28, 2022.<br>The dose expansion phase enrolled starting on January 5, 2021 and 3 patients are ongoing. The dose-expansion phase data cutoff date was June 19, 2023.<br>The ARC-8 study was conducted at 18 sites in the United States.
Outcomes	The protocol-specified primary objective was to assess the safety and tolerability of quemliclustat combination therapy in participants with advanced gastrointestinal malignancies. Secondary objectives included determination of the clinical activity of quemliclustat combination therapy in participants with advanced GI malignancies.<br>Tumor response was assessed by investigators using RECIST v1.1. Endpoints included objective response rate based on confirmed and unconfirmed best overall response, progression-free survival and overall survival. Safety data included type, incidence, seriousness, causality and severity of TEAEs and serious adverse events, as assessed by investigators according to the National Cancer Institute Common Terminology Criteria for Adverse Events, version 5.035. Adverse events were coded using the MedDRA v23.0.

Plants

Seed stocks	n/a
Novel plant genotypes	n/a
Authentication	n/a
